# Exploring the Potential for Yttrium Recovery from Secondary Sources: (Bio)hydrometallurgical and Solvometallurgical Routes

**DOI:** 10.3390/ma19132788

**Published:** 2026-07-01

**Authors:** Ewa Rudnik

**Affiliations:** Faculty of Non-Ferrous Metals, AGH University of Krakow, Mickiewicz Ave. 30, 30–059 Krakow, Poland; erudnik@agh.edu.pl

**Keywords:** yttrium, phosphors, phosphogypsum, red mud, coal fly ash, leaching

## Abstract

Yttrium is one of the lesser-known critical elements, but it has recently gained significant market attention due to a dramatic price increase of up to 1400% in Europe. Although its primary application is in phosphors (e.g., in LEDs), modern society heavily depends on these technologies, making yttrium indispensable. However, the limited availability of yttrium raises concerns about its long-term supply. Therefore, there is a need for efficient techniques to recover yttrium from secondary materials to ensure a stable supply. While the wastes contain only trace amounts of yttrium and often have complex elemental compositions, they are more readily available than primary sources. The yttrium content ranges from a few percent in spent phosphors to several hundred ppm in red mud, around a few dozen ppm in phosphogypsum, and up to several ppm in coal and coal fly ashes. Although conventional hydrometallurgical methods are commonly used, they lack selectivity for yttrium recovery. In contrast, unconventional solvometallurgical and bioleaching approaches currently play a relatively minor role in recovery applications. This review discusses a range of methods investigated for yttrium recovery from different types of secondary resources, including pretreatment (where applicable), leaching, and subsequent yttrium recovery from the resulting leachates. Although the chemical and phase compositions of yttrium-bearing waste materials differ substantially, necessitating tailored treatment strategies, acid leaching remains the predominant extraction route and is most commonly followed by solvent extraction and/or oxalate precipitation. Most studies reported to date have been conducted at the laboratory scale. Despite progress and the development of promising recovery concepts, the efficient separation of high-purity yttrium from other rare earth elements and co-existing impurities continues to represent the key obstacle to commercial-scale application.

## 1. Introduction

Yttrium (Y) was first discovered in the Swedish mineral ytterbite (gadolinite) in the late 18th century [1]. Its identification initiated a series of subsequent findings of several new elements within the same mineral, including erbium (Er), terbium (Tb), holmium (Ho), dysprosium (Dy), thulium (Tm), ytterbium (Yb), and lutetium (Lu). Today, yttrium, scandium (Sc), and the lanthanide subgroup constitute a set of 17 elements collectively classified as rare earth elements (REEs). Although yttrium (atomic number 39, atomic mass 88.9 u) is lighter than the lanthanides, it exhibits comparable physicochemical properties and a natural tendency to co-occur with heavy rare earth elements (HREEs; Gd–Lu) in minerals. This special yttrium behavior arises from values of atomic (180 pm) and trivalent ionic (104 pm) radii, which are particularly close to those of terbium, dysprosium, and holmium [2].

**Figure 1:**
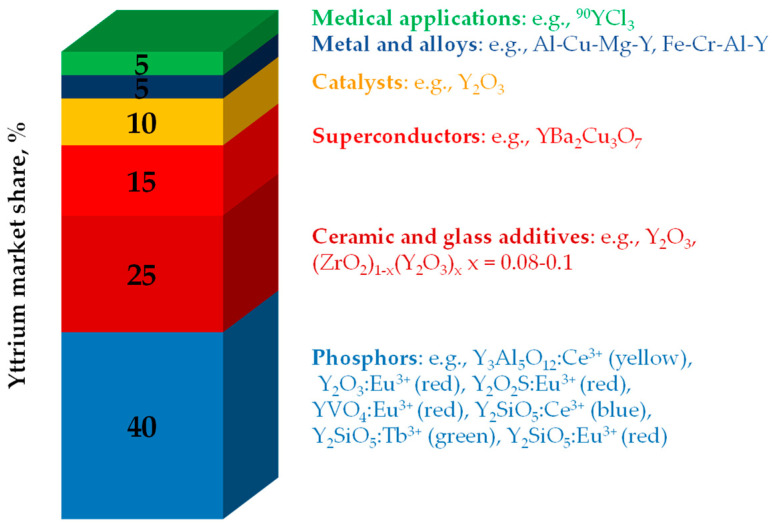
Global yttrium market distribution by end use [12] with main representative compounds and alloys [3,11].

Metallic yttrium is relatively soft (38–44 HB) and has a low density (4.47 g/cm^3^), while exhibiting relatively high melting (1526 °C) and boiling (3345 °C) points. It shows low tensile strength (150 MPa), moderate thermal conductivity (17 W/m·K), and electrical resistivity (59 nΩ·m; becoming superconducting at about −271 °C) [3]. These properties make yttrium an effective alloying element in lightweight, high-temperature alloys for strengthening aluminum and magnesium alloys [4,5] or for improving the high-temperature oxidation resistance of iron- and nickel-based alloys [6,7]. Nevertheless, yttrium compounds are of considerably greater technological importance than the pure metal (Figure 1). Among these, yttrium oxide (Y_2_O_3_) is the most important and accounts for the largest market [8]. It is forecast [8] that by 2033 the yttrium oxide market will reach near USD 525 million, representing a value three times that of yttrium metal and four times that of other yttrium compounds. The wide range of applications of yttrium oxide arises from its exceptional combination of properties, including long-lasting luminescence, a high melting point (2438 °C), thermal stability, high hardness (600–1000 HV), optical transparency over a wide wavelength range (0.23–9 μm), and a high refractive index (1.8–1.9 at 550–590 nm) [9]. Consequently, its main uses include phosphors for light-emitting diodes (LEDs), displays, fluorescent lamps (FLs), color television tubes; high-performance ceramics (yttria-stabilized zirconia (YSZ)) for aerospace and aircraft components, as thermal barrier coatings in gas-turbine engines and cutting tools; medical and dental implants; optical materials for lenses, mirrors, and glass products; high-power lasers (e.g., yttrium aluminum garnets, Y_3_Al_5_O_12_); catalysts in the petrochemical industry; high-temperature superconductors; YSZ electrolytes for solid oxide fuel cells (SOFCs); and production of the ^90^Y radiotracer for medical imaging (positron emission tomography (PET)), magnetic yttrium iron garnets (Y_3_Fe_5_O_12_) used in microwave and telecommunication devices, etc. [3,9,10,11].

These diverse high-technology applications of yttrium in both civilian and military sectors continue to drive its growing demand, with global consumption estimated at about 13,800 t in 2025 [13] compared with annual mine production of 10,000–15,000 t Y_2_O_3_ [14]. Nearly all yttrium production is concentrated in China (southern provinces: Fuijan, Guangdong, Jiangxi, Guangxi, Hunan) and Myanmar [14,15], where ion-adsorption clays constitute the principal source for the recovery of HREEs and yttrium [16]. Following the introduction of export licensing controls by China in April 2025 [17] and January 2026 [18], yttrium prices have risen sharply over the last year [14,15]. As a result, the price of high-purity yttrium oxide (99.999%) in Europe reached about USD 850/kg in February 2026, corresponding to an increase of more than 140-fold year-on-year [14]. Notably, the domestic Chinese market exhibits lower and relatively stable yttrium prices (USD 50–65/kg 99.999% Y_2_O_3_ [19]), as supply operates within a system under state influence [20].

Although yttrium sources and production outside China remain limited, alternative projects are increasingly being developed [14,21,22]. These include the identification and exploration of geological deposits in Sweden, Norway, Greenland, Kazakhstan, Australia, Canada, the United States, and African countries (Table 1). However, a key bottleneck remains the processing and separation of yttrium-bearing materials into high-purity products. In this context, USA Rare Earth reported in April 2026 the initiation of commercial production of metallic yttrium (99–99.5%) through its subsidiary Less Common Metals in the United Kingdom [23]. In recent times, however, access to more detailed data on the sources and production of rare earth metals has become increasingly limited, driven by rising geopolitical tensions, trade barriers, and concerns over resource security.

**Figure 2:**
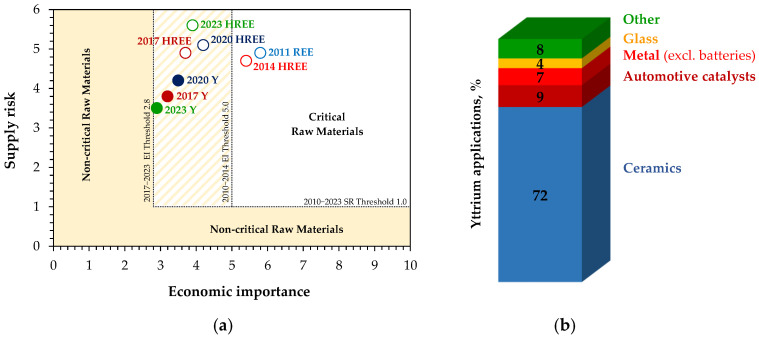
Significance of yttrium for the European Union: (**a**) position changes in the criticality matrix relative to the HREE group as a whole; (**b**) application distribution. Adapted from [34].

The dynamically evolving global situation resulted in yttrium being considered a critical and strategic material not only in the European Union [34], the United States [35], and China [36], but also in Australia, Brazil, Canada, India, Indonesia, and other countries [37]. The position of yttrium in the criticality matrices differs from that of the broader HREE subgroup when it is assessed as an individual element [34,38]. In the EU assessment (Figure 2a), its rather limited downstream demand profile (Figure 2b) results in somewhat lower economic importance than other HREEs, while its supply risk remains relatively significant due to its association with HREE processing streams.

While primary sources of yttrium remain predominant, secondary resources require greater attention, as most yttrium-containing products are discarded at the end of their service life without targeted recovery. As a result, yttrium recycling remains negligible [14,34,36], whereas a range of waste materials exhibits significant recovery potential, as demonstrated by laboratory-scale studies [11,39]. These include phosphors, phosphate-based waste, red mud, and less conventional sources such as coals and coal ashes. This highlights the need to develop strategies for yttrium recovery from secondary materials, particularly in light of current supply disruptions and significant price changes. Thus, the aim of the present work was to show (bio)hydrometallurgical and solvometallurgical approaches for yttrium recovery from various secondary materials, emphasizing their relevance to a sustainable circular economy [40].

## 2. Yttrium Recovery from Phosphors

### 2.1. General

Phosphors are chemical compounds that emit light upon excitation by photons or electrons from an external source [41,42]. These luminescent materials are widely used in light-emitting devices, such as fluorescent lamps, LEDs, and cathode ray tube (CRT) displays, as well as in radiation detectors, bioimaging, security markings, and optical sensing applications. Among these, the lighting and display sectors, driven primarily by white LED technology, account for the largest share of yttrium aluminum garnet Y_3_Al_5_O_12_ YAG commercial phosphors [43,44]. Other yttrium phosphors, such as Y_2_O_3_:Eu^3+^, YVO_4_:Eu^3+^, and Y_2_O_2_S:Eu^3+^, have been widely used in traditional CRTs and FLs [42].

The substantial share of LEDs (about 50%) and fluorescent lamps (about 13%) in the lighting product market (projected in 2025) [45], together with their yttrium content (Table 2) and lifetime (3 years for FLs, 11 years for LCD [46]), make end-of-life waste streams candidates for recycling and subsequent yttrium recovery [47]. This aspect is particularly important, as LEDs are not generally classified as hazardous waste, although they may contain potentially harmful elements like lead, arsenic, chromium, cadmium, and nickel [48,49,50], whereas fluorescent lamps are considered hazardous due to their mercury (6–21 mg per lamp [47]), lead, and copper content [50,51]. In contrast, CRTs from televisions and computer monitors are now largely obsolete and confined to niche markets, although about 40% of industrial and defense systems continue to use CRTs [52]. Though the volumes of waste CRT screens are declining, a significant fraction is not fully recycled and may be directed to landfill [53]. However, given the importance of yttrium and other rare earth elements such as europium, terbium, and gadolinium, appropriate treatment methodologies should be considered, as CRT waste is classified as hazardous due to the presence of leaded glass [54].

### 2.2. Waste Fluorescent Lamps

Fluorescent lamps typically employ calcium halo phosphate (Ca_5_(PO_4_)_3_(Cl,F):Sb^3+^, Mn^2+^), and three main types of REE-based phosphors—red-emitting (Y_2_O_3_:Eu^3+^), green-emitting (e.g., LaPO_4_:Ce^3+^,Tb^3+^ or (Ce,Tb)MgAl_11_O_19_), and blue-emitting (e.g., BaMgAl_10_O_17_:Eu^2+^)—to generate visible light [55,57]. These phosphors are mixed in various ratios [47,61,73] and account for 1.7–3% of total FL mass [47,74], representing the component with the highest market value compared to other constituents such as glass, plastics, and aluminum [47].

Phosphor powders are collected at the final stage of recycling processes for fluorescent lamps [47,75,76]. These processes typically involve the separation of components, including the removal of metal end caps, followed by the recovery of phosphors from the inner surfaces of the glass tubes, where mercury is subsequently removed by evaporation (81–89% Hg accumulates in phosphor powders [75]). Alternatively, entire lamps may be crushed (either under wet conditions or in a vacuum to capture mercury vapors), followed by sieving. In the former approach, the recovered phosphors are largely free of fine glass contamination, whereas the latter method often results in significant glass impurities (20–30% [74]). Some commercial processes have been developed for the further treatment of phosphor dust [47]. For example, Rhodia (currently Solvay Group), an only European company, commenced a project involving physical beneficiation followed by leaching in sulfuric and nitric acids, with subsequent precipitation of yttrium–europium oxalates (at the Saint-Fons and La Rochelle plants in France) [47,74,77]. The project was initiated in the 2010s, but further information on its operation is currently unavailable, likely due to proprietary constraints.

Although numerous laboratory-scale studies on yttrium recovery from FL phosphors have been conducted [47,51,75,76], research continues to focus on hydrometallurgical and solvometallurgical methods (Table 3), incorporating pretreatment stages in some cases [59,60]. It has been observed that red yttrium oxide-based phosphors are generally readily soluble in common acids during conventional leaching [57,73,78,79], while other types of phosphors, particularly green phosphors, require more rigorous conditions or preliminary processing (e.g., microwave-assisted acid leaching [79], sulfuric acid baking [80], alkaline roasting [61]) due to their more complex structure preventing easy release of other REEs. On the other hand, yttrium was found to be more concentrated in finer-grained FL powders. For example, Özkan et al. [79] showed that particles smaller than 45 μm contained 22% Y, while particles larger than 45 μm contained only 3.4% Y (at 15.7% Y in the total FL powder). In turn, Sinha et al. [81] reported significant variations in yttrium content across three fractions: 0.76% for 125–600 μm, 6.56% for 75–125 μm (31 wt% powder fraction), and 19.47% for particles below 75 μm (24% powder fraction).

The element can exhibit high leachability of around 98–100% during one-step leaching in strong inorganic acids such as hydrochloric HCl, sulfuric H_2_SO_4_, nitric HNO_3_, or methanesulfonic CH_3_SO_3_H of relatively medium concentrations [57,78,80]. During this leaching process, a fairly selective separation of yttrium (and europium) from other rare earth elements (Gd, Tb, La, Ce) is achieved, leaving most of them in the solid residue:

Y_2_O_3_:Eu^3+^ + 6H^+^ → 2Y^3+^ + Eu^3+^ + 3H_2_O (1)

Increasing the temperature [57,62], conducting microwave-assisted (MW) [62] or ultrasound-assisted [57] leaching, adding an oxidizing agent (e.g., hydrogen peroxide H_2_O_2_ [60]) to the leaching solution, or introduction of a pretreatment stage [59,60] does not significantly affect the leachability of yttrium compounds, but only influences the recovery efficiency of other REEs.

Similar recovery trends for yttrium are observed when leaching with organic reagents such as acetic acid CH_3_COOH [57,83], citric acid C_6_H_8_O_7_ [83], or in an acidic glycine C_2_H_5_NO_2_ solution [83]. Although organic acids may appear to be a more eco-friendly alternative to inorganic acids, Da Silva Alvarenga et al. [83] concluded that organic acids are not necessarily more environmentally favorable due to the significant impact of reagent production and electricity consumption, which affect climate change and human toxicity categories. The assessment also took into account the subsequent stages of yttrium recovery by precipitation of oxalate with an excess of oxalic acid, where the process efficiency was lower for nitrate- and acetate-based leachates (below 30%) compared to citrate- and glycinate-based solutions (50–80%). Notably, precipitation of yttrium oxalate appears to be the most common method for obtaining the final product, as it can be easily converted into oxide through simple heat treatment [80,82,84,85].

Leaching of yttrium from fluorescent lamp powders is not fully selective, as it depends on the acid concentration, with lower concentrations favoring higher selectivity [62,73,78,81]. As a result, the solution contains not only yttrium ions but also ions of iron, aluminum, calcium, phosphorus, europium, and other rare earth elements. This necessitates selective purification to obtain a high-purity yttria final product. The most commonly used method for yttrium separation is solvent extraction (SX) [78,82,84,85]. This technique exploits the uneven distribution between two aqueous and organic immiscible liquid phases and enables the organic phase to be recycled in a nearly closed loop through a combination of extraction and stripping operations conducted in separate stages. Bilen et al. [82] conducted a series of tests with various extractants, including D2EHPA, Cyanex 272, Cyanex 572, Cyanex P23, and Aliquat 336, to separate metal ions from nitrate leachate. With D2EHPA, recovery of yttrium and europium ions reached 98% and 95%, respectively. For the other extractants, the recovery of these two elements was below 35%. The extraction of the remaining ions (Ca, Tb, La, Ce, Gd, Al) was typically below 20% (except for Tb, which was below 30%). Similar results for D2EHPA were reported by Delice et al. [73]. In turn, Pavón et al. [84] investigated the separation of yttrium and europium ions from a diluted chloride solution. No separation (100% extraction) was achieved with standard extractants such as Cyanex 572 and D2EHPA or ionic liquid extractants like Cyphos 104, Primene 81R–Cyanex 572, and Primene 81R–D2EHPA. For other extractants, such as TBP, TOA, and Aliquat 336, extraction of both ions was under 10%. However, Cyanex 923 exhibited a noticeable difference, albeit with relatively low recovery of about 5% for europium and 30% for yttrium. The differences became more significant with higher extractant concentrations, reaching a maximum yttrium recovery of 80% (with europium recovery remaining below 10%). Tunsu et al. [78] also reported large separation factors for Cyanex 923, indicating its potential for separating Y, Tb, Eu, and Gd from Ce and La. In all cases, 4 M HCl was selected as the stripping agent to achieve total yttrium recovery from the loaded organic phases.

Finally, it is worth highlighting the less conventional approaches for yttrium recovery from FL phosphors. Rodriguez Rodriguez et al. [85] proposed combining hydrometallurgical and solvometallurgical operations using methanesulfonic acid. The process involved using pure acid for calcium-rich halophosphate dissolution in the first step, followed by application of a 5% acid solution for leaching yttrium and europium from the residue in the second step, and then using pure acid again in the third step for lanthanum leaching. The final rare earth element recovery was suggested through D2EHPA solvent extraction, followed by precipitation of oxalates.

An interesting method involves the use of aqueous two-phase systems ATPS developed by da Silveira Leite et al. [86]. ATPS are formed by mixing two aqueous solutions with distinct properties (a polymer and an electrolyte) under specific conditions of temperature, pressure, and concentration. Following phase separation, the system forms two phases: a polymer-enriched top phase and an electrolyte-rich bottom phase. In their study, fluorescent powder was initially leached with H_2_SO_4_–H_2_O_2_ solution, followed by treatment to precipitate REE oxalates, leaving the yttrium-reach solution (381 mg/L Y, 14 mg/L Eu, 21 mg/L Ca). The latter was then mixed with a polymer solution composed of L64 (triblock copolymer), Na_2_SO_4_, alizarin red, and water. The extraction of yttrium was 25% in the first stage, but after four subsequent stages, the yttrium extraction into the top phase increased to 90%. While this liquid–liquid extraction was reported as successful, no final yttrium product or recovery method was proposed.

### 2.3. Waste LED Modules

**Figure 3:**
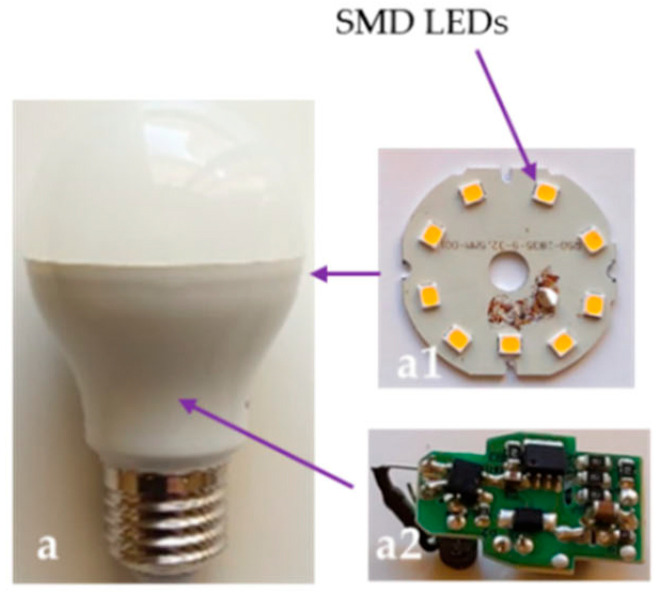
LED lamp (**a**) with SMD LEDs (**a1**) and driver (**a2**). Reproduced from [65] under License CC BY.

The adoption of LED lamps has surged over the last decade, with market penetration increasing from about 2% in 2012 to 76% in 2025 [87]. This shift has positioned LEDs as a more energy-efficient alternative to traditional incandescent and fluorescent lamps. However, unlike these older technologies, LED production requires the use of nearly a hundred different raw materials [88]. The complex material structure of LEDs means that recycling involves disassembling the components to separate fractions such as metals, plastics, glass, ceramics, drivers, LED modules, and filament LEDs [65]. From a rare earth element recovery perspective, these elements are particularly concentrated as phosphors in surface-mounted device (SMD) LEDs (Figure 3). Yttrium-containing commercial phosphors include, (yellow), YAG (yellow), YAG:Ce,Gd (yellow), Y_2_O_3_:Eu^3+^ (red), and Y_2_O_2_S:Eu^3+^ (red).

Due to the fact that waste LED streams are relatively new, the identification of element content is primarily characterized (Table 2), while recovery processes using solutions are still in the early stages of development. In fact, these methods are focused on a recovery of base metals (Au, Ag, Ga, In, Cu, Ni) than REEs [89].

Balinski et al. [64] proposed two methods for the selective liberation and concentration of valuable components in LED packages. Their study revealed that chemical treatment (soaking) with sodium hydroxide, hydrogen peroxide, acetic acid, naphthenic acid, isopropanol, ethylene glycol, and kerosene was unsuitable for the task. In contrast, solvents like acetone, 1-methoxy-2-propanol acetate, ethylbenzene, and toluene led to partial detachment of the LED encapsulation. Among these, toluene treatment produced the most promising results after one week. The enrichment factor for yttrium in the chemical treatment reached a notable value of 119 (4.6% Y). On the other hand, while thermal treatment proved ineffective for yttrium enrichment (with a factor of 6.2 and 0.34% Y), it facilitated the separation of aluminum.

Bourlinos et al. [90] introduced a microwave-assisted technique at the pretreatment stage for the thermal decomposition of plastic components into brittle and charred residue. Subsequent calcination (800 °C, air atmosphere) of the charred material (a mixture of charred lenses and LED chips) devoid of metallic pins (Fe, Ni, Ag) resulted in a product containing critical elements such as Ga, As, In, Y, and Au. These elements were then leached in two steps using concentrated acids: aqua regia followed by hydrochloric acid. Although the yttrium recovery in the first stage was only 10%, the second stage achieved complete dissolution, with the initial element content in the leached material being 0.24%. Microwave pretreatment was compared with conventional calcination, and though qualitative differences in the products were shown, but leaching results were not provided.

De Oliviera et al. [66] developed a method involving alkali fusion of separated LED devices, followed by leaching with nitric acid (1–4 M, 90 °C). The thermal pretreatment (700 °C, 3 h) with NaOH was aimed at converting PDMS encapsulate polymer and the hardly leachable aluminate phosphors into more soluble compounds:

PDMS + O_2_ → cyclic oligomers (2)

PDMS/cyclic oligomers + NaOH → Na_2_SiO_3_ + CO_2_ + H_2_O (3)

2Y_3_Al_5_O_12_ + 10NaOH → 10NaAlO_2_ + 3Y_2_O_3_ + 5H_2_O (4)

The combination of pretreatment-leaching experiments revealed that the best NaOH:LED ratio of 1:1 resulted in 83% yttrium extraction with 2.5 M acid. Further optimization of the leaching process achieved 91% yttrium extraction within 20 min, yielding a solution containing only 159 mg/L of yttrium compared to higher levels of contaminants (5 g/L Fe, 2.9 g/L Cu, 1.9 g/L Pb, 1.8 g/L Al, 1.1 g/L Zn). However, a specific recovery method was not presented, with the authors suggesting only solvent extraction as a potential solution.

More recent studies reported the application of deep eutectic solvents (DESs). Li et al. [91,92] used a choline chloride–malonic acid (ChCh–MA) (1:2) system at the leaching stage. The entire procedure involved the following steps: (1) collection of red phosphor from waste LED devices using focused ultrasound, (2) pyrolysis of the phosphor to separate it from the epoxy resin (300 °C, 3 h, nitrogen atmosphere), and (3) leaching with the ChCh–MA solvent. The leaching process was conducted in four different modes: using a bottom-focused microwave reactor (preceded by mechanical activation of the phosphor), conventional mechanical oscillations, ultrasound-assisted leaching, or focused ultrasound leaching. It was found that focusing microwaves or ultrasound on the leaching reactor significantly improved yttrium leachability with increased power, reaching a minimum of 90% at optimal conditions. Noteworthily, it was also observed [92] that adding a small amount of water to a DES bath improved phosphor dissolution, increasing the efficiency of microwave-assisted leaching from 20% in a nonaqueous system to 95% with 7.5% water content (Table 4). This enhancement was mainly attributed to the local generation of higher temperatures and vigorous agitation, which had destructive and dispersive effects on the leached particles, enhancing their contact with DES leachate and reducing the activation energy of the process. Although focused microwave or ultrasound leaching requires a higher power density than conventional processes, the reduction in leaching time results in lower overall energy consumption. The final recovery of yttrium from the DES was achieved by precipitation of yttrium oxalate using anhydrous oxalic acid, resulting in total recovery with a 50% excess of the reagent at 70 °C [91]. Further calcination produced yttrium oxide. The separation of the yttrium precipitate from the solution also enabled the recovery of the DES for reuse, with the key being the correct selection of the stoichiometric amount of oxalic acid to regenerate the leaching agent without additional operations.

### 2.4. Waste CRTs

Although CRT monitors are being gradually replaced by flat-screen technologies such as LCDs, LEDs, and OLEDs, a significant number of older displays remain in stock and are being collected for recycling. On average, 60% of the mass of a CRT display is glass, with only the front panel (which constitutes 65% of the total glass) covered by a phosphor powder containing REEs, while the back panel is made of leaded glass [93]. The luminescent layer typically consists of Y_2_O_3_:Eu^3+^ or Y_2_O_2_S:Eu^3+^ red-emitting phosphors, which are doped with ZnS:Cu, Al, or ZnS:Cu, and Au, Al, and ZnS:Ag compounds to produce green and blue colors, respectively [53]. To enhance the colors, the phosphor is often coated with iron oxide Fe_2_O_3_, cobalt blue Al_2_CoO_4_, or ultramarine blue Na_3_[SiAlO_4_]_6_·(S_3_)_2_. Consequently, CRT phosphors (1–7 g per complete CRT screen glass [71]) contain 13–19% Y, 0.5–2% Eu, 24–36% Zn, and 7–20% S [53]. Lin et al. [94] conducted a sequential extraction of CRT phosphor and found that 76% of the yttrium is bound to organic matter, 22% is associated with a scarcely soluble residue, while the remainder is distributed within carbonate and Fe/Mn oxide fractions, with no ion-exchangeable forms of yttrium detected.

**Figure 4:**
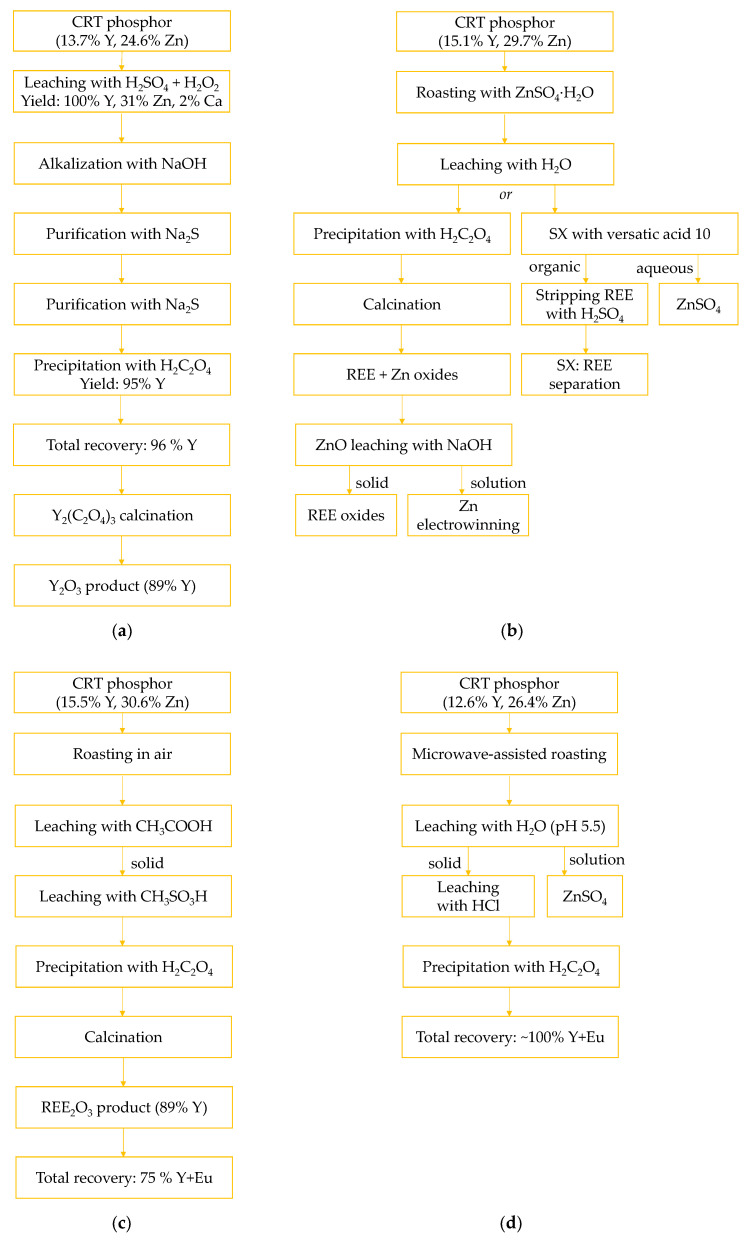
Schemes of yttrium recovery from CRT phosphors. Adapted from: (**a**) [95,96], (**b**) [67], (**c**) [97], (**d**) [70].

Recently, Figueiredo et al. [53] presented a summary of dismantling techniques used for phosphor collection and hydrometallurgical investigations for the recovery of yttrium and europium from CRT tubes. Thus, referring the reader to their work, only exemplary methods are presented here to show demonstrate their routes and efficiencies (Figure 4).

The primary challenge in the hydrometallurgical treatment of CRT phosphors lies in their sulfide nature, which requires the use of strong, non-oxidative inorganic acids. This, however, results in the release of harmful gases, including the toxic hydrogen sulfide H_2_S [68,94,98].

Y_2_O_2_S + 3H_2_SO_4_ → Y_2_(SO_4_)_3_ + 2H_2_O + H_2_S (5)

ZnS + H_2_SO_4_ → ZnSO_4_ + H_2_S (6)

This issue can be mitigated by introducing an oxidizing agent (e.g., H_2_O_2_) into the acid leaching solution, which converts sulfide ions into sulfur [72] or SO_2_ [68], thereby reducing the release of toxic gases.

2Y_2_O_2_S + 6H_2_SO_4_ + 2H_2_O_2_ → 2Y_2_(SO_4_)_3_ + 8H_2_O + S_2_ (7)

ZnS + H_2_SO_4_ + 3H_2_O_2_ → ZnSO_4_ + 4H_2_O + SO_2_ (8)

Miskufova et al. [68] showed that under the same conditions (0.25–1 M H_2_SO_4_, 80 °C, L/S ~20), yttrium leaching reached only 4–8% after 1 h, whereas in the presence of H_2_O_2_, it increased to 60–90% within just 10–20 min. Notably, better results were achieved in moderately concentrated acid solutions (0.25–0.4 M) compared to both more diluted and highly concentrated solutions. This concentration effect was further investigated in other studies [72], though no significant impact was observed. Additionally, raising the temperature from room temperature to 80 °C enhanced yttrium leachability, a finding also confirmed by other researchers [69,72].

Lie and Liu [69] compared different leaching approaches using only H_2_SO_4_ as the leachate. They reported that closed-vessel microwave leaching (125 °C) could achieve 90% yttrium recovery from CRT powder within 1 h. In contrast, atmospheric pressure leaching (400 W, 105 °C) reached the same level after 20 min longer, while conventional heating leaching (105 °C) resulted in 80% yttrium recovery after 3 h. These differences were attributed to the accelerated heat accumulation in the closed-vessel system, whereas in the other two systems, only a portion of the heat was utilized for the reaction, with the rest dissipating into the surroundings.

In turn, Resende and Morais [98] performed digestion (25 °C, 15 min) of ground CRT screens with concentrated H_2_SO_4_ to convert sulfides into sulfates, although H_2_S was released during the process. The digestion product was then water-leached, achieving 98% dissolution of yttrium and europium within 1 h. However, no further separation method was proposed.

Alternatively, roasting pretreatments can be used to prevent the formation of H_2_S during subsequent leaching. However, these processes are energy-intensive due to the requirement for high temperatures (600–1000 °C). Forte et al. [97] focused on the smallest particle fraction of CRT powder (<450 µm), as it contained the majority of the phosphor. When roasted in an air atmosphere, sulfide ZnS was converted into oxide ZnO, achieving up to 79% conversion at 800 °C, whereas only 51% ZnO was obtained at 1000 °C. At higher temperatures, ZnS remained in trace amounts, with 20% converting to silicate Zn_2_SiO_4_ and 27–30% forming other zinc compounds. The optimal roasting temperature was determined to be 850 °C, yielding 73% zinc as oxide. The roasted material was then leached in two steps (Figure 4c): first with acetic acid to remove zinc, followed by using methanesulfonic acid to dissolve the rare earth elements, which were subsequently recovered as oxalates (yttrium-europium oxides) in the final stage.

Önal and Binnemans [67] converted sulfides from CRT into water-soluble sulfates by roasting with zinc sulfate monohydrate (600–900 °C). Two routes were then proposed to separate zinc from rare earth elements (Figure 4b). The first route, based on classic oxalate precipitation, was followed by alkaline leaching of zinc to produce electrolyte for metal electrowinning. The second route employed solvent extraction with versatic acid 10 for selective REE separation. However, the first method was ultimately recommended as more conventional and straightforward. In contrast, Li et al. [74] developed an alternative method for converting powders into sulfates using a low-temperature treatment (55–95 °C) with concentrated H_2_SO_4_. The product was then leached with water (35–95 °C), with a 10 min sulfatization process being sufficient to achieve total yttrium leachability. Srivastava et al. [70] proposed a different method for converting powders into a sulfate and oxide mixture using microwave roasting (600–800 °C) with sulfuric acid. The product was then leached with various acids, with HCl being selected as the best leachate compared to H_2_SO_4_ and HNO_3_. Under optimal leaching conditions (2 M HCl, 90 °C, 1 h), 99% leaching efficiency was achieved for the product roasted at 800 °C. The final metal recovery was performed using the classic oxalate precipitation method (Figure 4d).

A notable study was reported by Lin et al. [94], who compared conventional acid leaching (0.5 M H_2_SO_4_, 25–65 °C) with subcritical water extraction SWE (pressure 10 kg/cm^2^, 300 rpm, acid modifiers, nitrogen atmosphere). Under conventional leaching, up to 30% of yttrium was maximally dissolved from the CRT phosphor (0.0044% Y). In contrast, the efficiency of SWE was strongly dependent on the acid modifier (0.5 M), with yttrium recovery of 2.6% for HCl, 22.3% for H_2_SO_4_, and 22.1% for HNO_3_. Since sulfuric acid showed the best selectivity for inhibiting zinc and lead dissolution, this modifier was further optimized for yttrium recovery. Under optimal conditions (0.75 M, 150 °C), complete yttrium dissolution was achieved within 0.5 h.

## 3. Yttrium Recovery from Phosphogypsum

### 3.1. General

Phosphogypsum is a primary by-product of phosphate fertilizer production [99]. It is formed during the production of phosphoric acid H_3_PO_4_ from raw fluorapatite Ca_10_(PO_4_)_6_F_2_ treated with concentrated sulfuric acid:

Ca_10_(PO_4_)_6_F_2_ + 10H_2_SO_4_ + 20H_2_O → 6H_3_PO_4_ + 10CaSO_4_·H_2_O + 2HF (9)

On average, the production of 1 t H_3_PO_4_ results in the generation of 3.5–5 t phosphogypsum. It is estimated that 280–300 million tons of this waste are manufactured globally each year [100,101], with China (81 Mt/y), the USA (30 Mt/y), and Morocco (15 Mt/y), the main producing countries [102]. About 85% of the waste is either stored in stockpiles (over 830 million t accumulated) near fertilizer manufacturing plants or discharged into water bodies [101], while the remaining part is utilized in in building materials, agriculture or in cement production [102]. Calcium sulfate dihydrate is the main waste component (85–95%), and although phosphogypsum is not classified as hazardous waste, it poses potential risks due to the presence of radioactive elements (up to 0.01% ^226^Ra, ^232^Th, ^235^U, ^40^K) and the release of toxic substances such as fluorine (0.05–1.85%) or heavy metals (below 1% As, Pb, Cd, Cr, etc.) [99,103].

Phosphogypsum also contains notable concentrations of rare earth elements (0.005–0.6 wt%) [99,103,104] accumulated (98%) in gypsum and monazite (phosphate) [105]. The yttrium content in this waste is variable, but it often constitutes significant share of the total REE (Table 5). A sequential analysis conducted by Guan et al. [106] demonstrated that REE predominantly accumulate in the metal oxide (39%), residual (31%), and organic matter (18%) fractions, with the remainder distributed between ion-exchangeable (7%) and carbonate (5%) forms. Notably, the distribution of yttrium follows a similar trend. Among these, ion-exchangeable, carbonate, and metal oxide forms exhibit higher solubility under relatively weak acidic conditions. In comparison, calcium (the main element of phosphogypsum) accumulates predominantly in the residual (56%) and organic matter (20%) fractions, indicating that a significant portion of yttrium is incorporated into the gypsum lattice and metal oxide structure. In turn, Qing et al. [107] identified three principal modes of REE occurrence in phosphogypsum: as mineral inclusions (e.g., xenotime, monazite), as isomorphic substitutions for Ca^2+^ within the gypsum lattice, and as dispersed soluble salts, all of which influence their release during leaching.

Phosphogypsum is generally recognized as a promising secondary source of rare earth elements, and numerous laboratory-scale studies have examined the leaching behavior of the entire REE group [99,100,102,103,104]. In most cases, however, the obtained products are mixed concentrates containing multiple elements. Large-scale tests (sulfuric acid leaching combined with separation methods like solvent extraction, ion exchange, precipitation) have been also explored to produce concentrate final products, as in Poland (a 10–40% REE concentrate) [121] or Russia in 2010s (a 60% REE oxocarbonate concentrate) [122]. The absence of commercial-scale implementation for REE recovery from phosphogypsum reflects not only technological limitations but, above all, economic constraints associated with these processes [100,103]. However, noteworthy progress has been made in South Africa: Phalaborwa Rare Earths developed a project [123,124] where leaching is combined with ion exchange and solvent extraction to obtain high-grade mixed rare earth products, including Nd–Pr oxides and mixed carbonates (enriched in Sm, Eu, Gd, Y, Tb, and Dy). The project is currently at the stage of preparing a definitive feasibility study and is expected to commence operations in 2028. The planned annual output includes 1850 t Nd_2_O_3_–Pr_2_O_3_, 140 t Y_2_O_3_ and 80 t Dy_2_O_3_–Tb_4_O_7_. The operation is designed to process roughly 2.2 million tons of phosphogypsum per year over an estimated period of 16 years. Finally, it is worth mentioning that the large-scale examples of REE extraction from phosphogypsum report overall recovery rates in the range of 63–65% [122,124].

### 3.2. Hydrometallurgical Treatment

Direct leaching of phosphogypsum is most commonly performed using inorganic acids (Table 6). Considering the identified modes of yttrium occurrence in this material [105,107], its extraction efficiency may be constrained by limited release from the CaSO_4_·2H_2_O matrix. On the other hand, the use of leaching agents should be controlled to avoid unnecessary dissolution of gypsum, which can be recovered as a valuable by-product (hydrated or anhydrite calcium sulfate) [107,116,121]:

xCaSO_4_·REE↓ + yH_2_SO_4_ → xCaSO_4_↓ + zREE^3+^ + yH^+^ + ySO_4_^2−^ (10)

xCaSO_4_·REE↓ + yHR → (x − n)CaSO_4_↓ + nCa^2+^ + zREE^3+^ + yH^+^ + yR^−^ (11)

where R is NO_3_^−^ or Cl^−^. Taking this into account, Li et al. [125] reported that the solubility of CaSO_4_ in acidic solutions (0.5–2.5 M, 45–85 °C) generally follows the order H_2_SO_4_ < HCl < HNO_3_. In the case of HCl, a clear increase in solubility was observed with increasing acid concentration.

**Table 6:** Yttrium leaching from phosphogypsum.

Y Concentration, ppm	Leaching Conditions	Leaching Efficiency, %	Ref.
129	0.5 M H_2_SO_4_, 25 °C, 8 h, S/L 5%	61	[116]
	3 M HNO_3_, 25 °C, 8 h, S/L 5%	84	
120	2.5 M H_2_SO_4_, 85 °C, 0.3 h, S/L 3%	52	[125]
	2.5 M HNO_3_, 85 °C, 0.3 h, S/L 3%	85	
	2.5 M HCl, 45 °C, 0.3 h, S/L 3%	99	
74	1.65 M HNO_3_, 80 °C, 1 h, S/L 10%	65	[126]
	1.65 M HCl, 80 °C, 1 h, S/L 10%	88	[106]
90 Y_2_O_3_	4 M H_2_SO_4_, 30 °C, 3 h, S/L 25%	64	[107]
192	2 M H_3_PO_4_, 25 °C, 4 h, S/L 12.5%	75	[114]
	H_2_O, pH 3 (H_2_SO_4_), 4 h, S/L 12.5%	70	
163	2 M HCl, 55 °C, 2 h, S/L 12.5%	63	[112]
	3 M CH_3_SO_3_H, 25 °C, 2 h, S/L 12.5%	84	
	p-CH_3_C_6_H_4_SO_3_H, 25 °C, 2 h, S/L 12.5%	62	
120	1.5 M HCl, 45–85 °C, 1 h, S/L 7%	69–85	[127]
		92–99.5 *	
54	3 M HCl, 3–5% NH_4_Cl, 25 °C, 1 h, S/L 10%	70%	[119]

* Microwave-pretreated (1200 W, 15 min) phosphogypsum.

Yttrium appears to exhibit relatively favorable leachability in mineral acids compared to other rare earth elements [106,107,109,114,126]. Cánovas et al. [116] investigated the acid leaching of yttrium-rich phosphogypsum. The highest recovery was achieved using 3 M HNO_3_, yielding 84% yttrium extraction (on average 84 ± 2% for total REEs), although this was accompanied by the dissolution of 63% of the initial gypsum content. In contrast, leaching with 0.5 M H_2_SO_4_ resulted in 61% yttrium recovery (52 ± 6% for total REEs) while limiting gypsum dissolution to below 6%. The introduction of a water pretreatment step enabled the removal of selected impurities (80% of Mg, Mn, and As; 40% of Fe; and 30% of Cd and Zn) without any loss of REEs. This approach creates the possibility of obtaining gypsum suitable for applications such as fertilizers, as it meets relevant impurity standards. The final products of the process were aqueous solutions, but no subsequent method for REE separation was proposed.

Li et al. [125] used more concentrated acids (2.5 M) for the dissolution of yttrium, along with Dy and Nd. Rapid leaching kinetics were observed for HCl and HNO_3_, with metal dissolution reaching stable levels within 5 min, whereas in the case of H_2_SO_4_, four times that was required. For all acids, increasing the liquid-to-solid ratio and temperature had a positive effect on leaching efficiency. A higher acid concentration enhanced metal dissolution, although no significant improvement was observed for H_2_SO_4_ due to low solubility of calcium and REE sulfates (probably originated from common ion effect and/or double sulfate salt formation). Based on the overall results, HCl was identified as the most effective leaching agent, achieving up to 62–99% yttrium recovery. It was shown that during leaching, the acids disrupt the phosphogypsum structure, releasing REEs entrapped within it as separate phases (e.g., yttrium as Y_2_O_3_ and Y_2_(SO_4_)_3_). Therefore, the breakdown of the crystal lattice plays a crucial role in achieving high leaching efficiencies.

Qing et al. [107] explored the same mineral acids at higher concentrations (4 M), reporting increased yttrium recovery (62–65%) in the order HNO_3_ < H_2_SO_4_ < HCl. However, taking into account the modes of REE occurrence and the fact that the gypsum matrix undergoes recrystallization during leaching facilitating the release of soluble REEs into the aqueous phase, the authors identified H_2_SO_4_ as a promising leaching agent. They proposed a wastewater-free recovery scheme (achieving 53% REE recovery in a five-stage cycle), based on leaching in relatively dilute acid (0.5–2 M), followed by precipitation of REE oxalates (converted then to oxides). This was combined with wet screening of the solid residue from leaching stage, enabling the separation of REE-bearing phases from a high-purity gypsum product (95%). Although wet screening led to the accumulation of REEs in specific grain size fractions (36% in >200 mesh and 37% in <500 mesh), no comparable enrichment of yttrium was observed across the fractions. This was likely due to its low initial content in the raw material (90 ppm Y_2_O_3_, corresponding to about 1.8% of total REO).

Brahim et al. [112] compared the leachability of REEs using conventional hydrochloric acid and two less common reagents, methanesulfonic acid CH_3_SO_3_H and p-toluenesulfonic acid p-CH_3_C_6_H_4_SO_3_H, selected as more environmentally friendly alternatives. Among the tested reagents, CH_3_SO_3_H exhibited the most favorable performance, achieving total REE leachability of 78% while maintaining low dissolution of the phosphogypsum matrix at room temperature. Under optimal conditions, yttrium recovery reached 84%, comparable to that of Ho, Tm, and Lu, although lower than for Sm, Eu, and Gd (94%).

Lambert et al. [127] evaluated the applicability of microwave pretreatment (800–1200 W) of phosphogypsum prior to acid leaching with HCl for the extraction of yttrium, along with Nd and Dy. This preliminary step enhanced yttrium recovery by about 20%, reaching over 92% at the highest applied microwave power. The improvement was attributed to thermal degradation of the phosphogypsum structure, which facilitated the release of REEs. This effect was associated with the gradual transformation of CaSO_4_·2H_2_O into less hydrated phases, namely hemihydrate CaSO_4_·0.5H_2_O and anhydrite CaSO_4_, with their proportion increasing with both microwave power and treatment time, reaching roughly a 1:1 ratio at 1000–1200 W (5–15 min).

To reduce HCl consumption during leaching and thereby limit its environmental impact, Jebali et al. [119] investigated the addition of ammonium chloride NH_4_Cl to acidic solutions in order to enhance the release of ion-exchangeable REE^3+^ ions (Y^3+^, Nd^3+^, La^3+^). The presence of the additive had a particularly pronounced effect on yttrium recovery, which increased to about 70%, representing a twofold improvement in 3 M HCl with 3–5% NH_4_Cl. At the same time, gypsum solubility was found to depend on ionic strength, adjusted through the addition of alkali chlorides NaCl and KCl. Consequently, the leaching efficiency followed the order NaCl > KCl > NH_4_Cl. This trend was attributed to enhanced CaSO_4_ dissolution, driven by a decrease in the activity coefficients of Ca^2+^ and SO_4_^2−^ ions as a result of intensified ionic interactions in solution.

Finally, a noteworthy example of an environmentally friendly REE enrichment approach was reported by Hammas-Nasri et al. [118]. The proposed procedure involved initial washing of phosphogypsum with a NaCl solution, followed by leaching using sodium carbonate Na_2_CO_3_. This two-step treatment generated two types of solid residues, in which yttrium concentration increased from about 81 ppm in the original phosphogypsum to 468 ppm after chloride washing and further to 528 ppm following carbonate treatment. Overall, yttrium enrichment reached nearly 85%, with a comparable average value observed for the total REE content. The obtained concentrate was then leached in a two-step process with 15% H_2_SO_4_ in a high-pressure and high-temperature (100 °C) reactor [117], producing an REE-rich liquor (4.3 g/L) containing 0.5 g/L Y^3+^. Subsequent recovery performed with ammonia (in three stages up to pH 6) resulted, with nearly 99% of the REEs incorporated into ammonium sulfate crystals. Final yttrium concentration in the solution after completed precipitation decreased to 0.001 g/L.

The presented examples of recovery indicate that effective treatment is generally achieved using strong acids. However, the final product is typically a mixed concentrate that still requires further separation into individual elements, which remains an area requiring additional research and process development.

### 3.3. Biohydrometallurgical Treatment

Bioleaching of phosphogypsum has been investigated for the recovery of rare earth elements [128,129,130,131,132,133,134,135]. Compared to conventional acid leaching, bioleaching relies on metabolites produced by microorganisms. In addition to biogenerated sulfuric acid by bacteria, organic acids produced by fungi exhibit strong potential due to their complexing properties.

Saolo et al. [129,130] employed *Desulfovibrio* bacteria to reduce sulfate to sulfide under anaerobic conditions in a continuous bioreactor. Leachates for the bioreactor were prepared by mixing phosphogypsum (36 ppm Y) with water, followed by acidification with sulfuric acid. Total REE dissolution in water was below 1%, whereas in the presence of acid (0.01–0.05 M) it increased to 6–62%. The average yttrium concentrations in the bioreactor influents were relatively low, with values in the order of 146–220 µg/L and yields of 8–10% in diluted acid systems (0.01–0.02 M). The bioreactor treatment achieved 97% sulfur removal and over 99% REE removal with the tolerable acid concentration limited to 0.01 M. The resulting bioreactor precipitate showed significant lanthanide further recovery processes (not developed). This material contained 86.5 ppm yttrium, predominantly (58%) associated with a single phase consisting of calcium phosphate-sulfate enriched in REEs with trace amounts of aluminum.

In contrast, Tayar et al. [131] investigated sulfuric acid media generated by two types of sulfur-oxidizing microorganisms. These were consortia derived from acid mine drainage and *Acidithiobacillus thiooxidans* (an aerobic, mesophilic bacterium). As the latter produced higher amounts of sulfuric acid, it was selected for more detailed studies on phosphogypsum treatment. The highest REE extraction (about 60%) was achieved via a two-step bioleaching process and at the reactor scale (3 L, S/L 10%, 60 days) total REE recovery reached 55%. Among the individual REEs, neodymium was the most readily leached (98%), whereas the average leachability of other elements 60% (62% Y). For comparison, REE extraction in single-step processes typically ranged from 17% to 30% (30% Y), except for holmium (50%).

Tong et al. [132] proposed a bioleaching process involving a co-culture of *Acidithiobacillus ferrooxidans* (an aerobic, mesophilic iron- and sulfur-oxidizing bacterium) and *Acidiphilum cryptum* (an aerobic, mesophilic heterotrophic bacterium). They demonstrated a significant synergistic effect arising from medium acidification driven by both proton release during jarosite formation and the production of organic acids by *A. cryptum*. Over a 30-day period, changes in solution concentrations were monitored for four REEs (Y, La, Nd, Ce) and the strongest synergistic effect was observed for yttrium: recovery in the co-culture system reached 70% compared to only 20–25% in single-bacterium systems. Under optimal conditions in a two-step leaching process, recovery reached 84% for yttrium, 85% for lanthanum, 70% for neodymium, and 39% for cerium.

**Figure 5:**
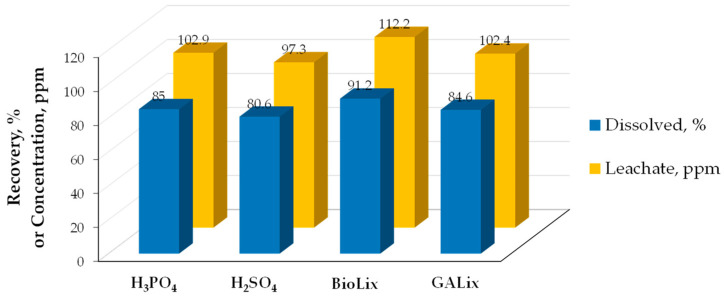
Effect of leaching agent on concentration and recovery of yttrium from synthetic phosphogypsum. Adapted from [133].

Antonick et al. [133] compared bio- and mineral-acid leaching of REEs from synthetic phosphogypsum doped with various elements (Y, Ce, Nd, Sm, Eu, Yb). The study employed two mineral acids (0.2 M H_2_SO_4_ and H_3_PO_4_) as well as acidic media (pH 2) based on gluconic acid, including a commercial reagent GALix and a biolixiviant BioLix. The latter was a spent culture medium derived from *Gluconobacter oxydans* (an aerobic, mesophilic acetic acid bacterium). Among all investigated single REE–phosphogypsum systems (with a general formula of CaREE_0.01_Na_0.02_(PO_4_)_0.01_SO_4_·0.5H_2_O), only yttrium showed broadly comparable leaching behavior across all media, whereas the other dopants were only weakly recovered in phosphoric acid. This discrepancy was not explained by the authors. The results demonstrated that at equivalent molar concentrations, the biolixiviant achieved higher REE extraction efficiency than gluconic and phosphoric acids, but it was less efficient than sulfuric acid (Figure 5). In contrast to the organic acids, the mineral acids did not facilitate REE extraction (at pH 2), likely due to differences in complexation behavior and reaction kinetics. It should be also noted that the behavior of synthetic model samples may differ from that of real industrial waste materials; however, the latter were not examined in this study.

**Figure 6:**
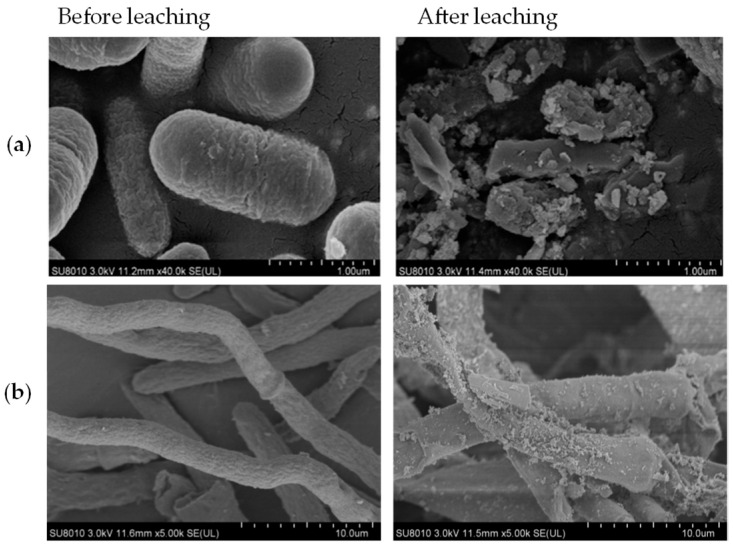
SEM images of microorganisms: (**a**) *Gluconobacter oxydans* [134]; (**b**) *Aspergillus niger* [135]. All images adapted under License CC BY.

Zhang et al. [134] employed a *Gluconobacter oxydans* strain (Figure 6a) for the extraction of rare earth elements from real phosphogypsum waste. Over 21 days, the total recovery reached 25% (18 mg/L), although no data were reported for individual REEs. Post-leaching analysis of the biomass indicated cell disruption, with effective adsorption of various REEs, among them Y, Ce, La, and Nd.

**Figure 7:**
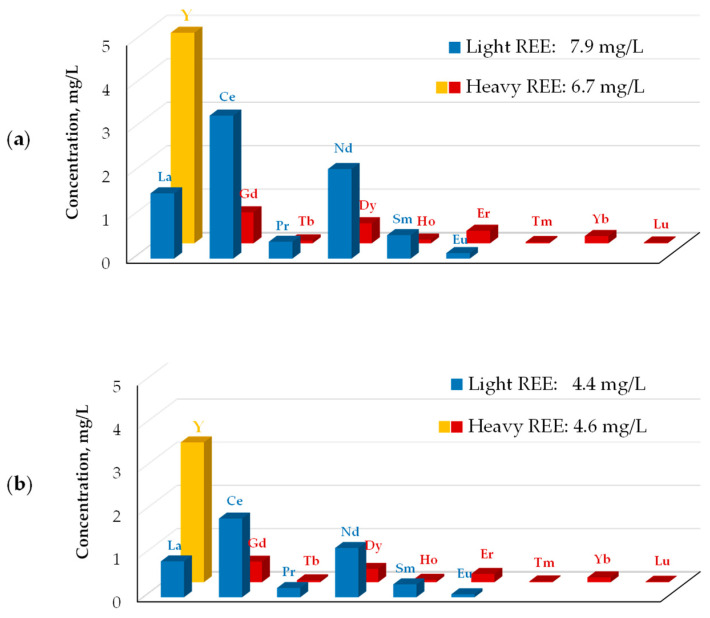
Effect of leaching type on recovery of yttrium and other REEs from phosphogypsum: (**a**) bioleaching with *Aspergillus niger*; (**b**) chemical leaching with a mixture of organic acids with a composition similar to the fermentation solution. Adapted from [135].

In other investigations, Zhang et al. [135] analyzed the applicability of *Aspergillus niger* (Figure 6b), a fungus known for producing a range of organic acids (citric, gluconic, oxalic, tartaric, and ketogluconic). The results demonstrated that bioleaching can markedly enhance overall yttrium (and other REEs) leachability when compared with chemical leaching using a synthetic organic acid mixture (Figure 7). Although no element-specific extraction efficiencies were reported, the observed trends suggest considerable potential for further application of this approach.

It should be emphasized that although the cited examples highlight bioleaching as a sustainable route for the solubilization of REEs from industrial waste and suggest its potential as a greener alternative, detailed demonstrations of subsequent recovery and separation processes from the resulting solutions are lacking.

## 4. Yttrium Recovery from Red Mud

### 4.1. General

Red mud is a reddish-brown, highly alkaline (pH 10–14) solid waste generated during alumina Al_2_O_3_ production from bauxite, predominantly through the Bayer process [136]. It has been estimated [137,138] that 0.8–2.5 t of red mud (typically 1.0–1.5 t) are produced per 1 t Al_2_O_3_, depending on the type of bauxite processed. In 2025, global red mud generation reached over 175 million tons [139], corresponding to an alumina output of 154 million tons [140]. The management and utilization of this waste remain a major global technical issue, as its utilization rate is estimated to be below 5% [141,142], with current applications largely limited to its use as an additive in construction materials [139]. Consequently, enormous quantities of this hazardous waste [136] continue to accumulate in disposal sites. The cumulative stockpile is estimated at 4 billion tons, making red mud one of the largest untapped secondary mineral resources worldwide [138,141].

The chemical composition of red mud [137] is highly complex and typically dominated by iron (5–60% Fe_2_O_3_), occurring mainly as goethite, hematite, and magnetite. Other major constituents include aluminum (5–30% Al_2_O_3_), present in the form of aluminosilicates, aluminates, gibbsite, and diaspore; calcium (2–14% CaO), associated with aluminates, aluminosilicates, and carbonates; titanium (up to 15% TiO_2_), primarily occurring as oxides; silicon (3–50% SiO_2_), mainly as aluminosilicates and silica; and sodium (1–10% Na_2_O), largely incorporated into aluminosilicate phases. Beyond these major components, red mud contains a broad spectrum of trace elements, including economically valuable metals such as gallium and rare earth elements. Among the latter, lanthanum and cerium may occur at concentrations reaching several hundred ppm [141]. The yttrium content in red mud varies considerably depending on the origin and mineralogical characteristics of the residue, typically ranging from several dozen to several hundred ppm (Table 7). Its concentration is higher than that found in the original bauxite ore due to the enrichment effect occurring during alumina extraction.

Couturier et al. [144] investigated the speciation of yttrium in red muds derived from different types of bauxite (lateritic and karstic), taking into account storage conditions (tropical or Mediterranean climate; open-air, covered, or wet storage) and accumulation periods ranging from 0 to 110 years. Their study demonstrated that yttrium concentration is influenced primarily by stockpile conditions, whereas its chemical form is governed mainly by the type of parent ore. In red mud originating from lateritic bauxite, yttrium occurred predominantly as xenotime phosphate, while in residues derived from karstic bauxite it was mainly adsorbed onto or incorporated within other mineral phases, particularly iron oxyhydroxides and hydroxyapatite minerals. In contrast, Vind et al. [145] identified yttrium-bearing phosphate phases in residue generated from karstic bauxite ore.

Classical sequential extraction procedures have likewise been applied to investigate yttrium occurrence in the red mud, however, the obtained results should be regarded as approximate, as these methods do not directly identify specific mineralogical phases. Using the Tessier sequential extraction procedure, Gu et al. [143] examined yttrium distribution in two diasporic red muds differing in iron content. About 50 ± 5% of yttrium was associated with the residual fraction, followed by around 10% bound to organic matter. Minor differences were observed in the carbonate-bound fraction, accounting for approximately 10% in high-iron red mud and about 3% in low-iron diasporic residue. Notably, yttrium was absent in both the water-soluble and ion-exchangeable fractions. Comparable observations were reported by Çelebi [150], who demonstrated that nearly 80% of yttrium accumulated in the residual, sparingly soluble fraction, while 10% was associated with reducible and oxidizable fractions, with no ion-exchangeable yttrium detected. These findings suggest that efficient yttrium recovery from red mud is unlikely to be achieved under mild leaching conditions.

### 4.2. Hydrometallurgical Treatment

The recovery of valuable metals from red mud has been the subject of extensive research, with most studies focusing primarily on the extraction of rare earth elements as a whole group or on scandium recovery [142,152], while comparatively less attention has been devoted specifically to yttrium. Nevertheless, a number of studies have investigated yttrium recovery through acid leaching using strong mineral acids (Table 8).

Red mud is an extremely alkaline material, which represents a significant disadvantage for acid leaching processes due to excessive reagent consumption associated with neutralization. Simple water prewashing has proven to be largely ineffective in reducing its alkalinity [146]. It was demonstrated that even after four consecutive washing cycles, the pH of the residue remained close to 10 due to the strong buffering capacity of alkaline solid phases present in the material. Moreover, only 10–15% of sodium was dissolved during the washing process, indicating that recovery of sodium hydroxide at this stage is not feasible.

Comparative studies performed on red mud samples with identical chemical and mineralogical compositions are of particular importance, as they enable evaluation of the actual influence of the leaching agent itself under conventional mode. Borra et al. [146] compared the efficiency of yttrium leaching using various diluted acids at concentrations up to 1 N and demonstrated that yttrium was among the most readily leached rare earth elements investigated (Y, Sc, La, Ce, Nd, and Dy). In general, relatively stable extraction efficiencies were achieved at acid concentrations above 0.2 N. The leaching efficiency remained below 70% and decreased in the following order of acids applied: HCl ≈ HNO_3_ (~70%) > H_2_SO_4_ ≈ CH_3_SO_3_H (~55%) > C_6_H_8_O_6_ (~50%) > CH_3_COOH (~20%). Although the use of more concentrated HCl solutions (1–6 N) improved overall REE extraction, with yttrium recovery reaching about 80%, this was accompanied by substantial dissolution of matrix components, including iron (~60%) and calcium (~100%), as well as aluminum, silicon, and titanium (30–50%). In turn, Karakaya et al. [152] optimized direct leaching conditions using the Taguchi experimental design. Under the optimal conditions, yttrium recovery increased in the following order: H_2_SO_4_ (1% at 75 °C) < HNO_3_ (98% at 95 °C) < HCl (100% at 75 °C). The very low leaching efficiency observed for sulfuric acid was associated with the presence of calcium compounds, which promoted gypsum precipitation, and silica polymerization under strongly acidic conditions. Among the investigated acids, HNO_3_ was ultimately identified as the most suitable leaching agent due to the highest overall extraction efficiencies achieved for the investigated REEs as a whole (74–87%).

Ebrahimi-Moghaddam et al. [149] compared the leachability of elements from red mud using a microwave-assisted leaching approach combined with a series of organic acids (malic, acetic, formic) as well as sulfuric acid. In the case of organic acid solutions, the extraction efficiencies were relatively low, but increasing acid concentration improved the results. For 0.6 M HCOOH, the highest yttrium yield reached 30% compared with 59% obtained in 1 M H_2_SO_4_. Interestingly, prolonging the leaching time led to a decrease in extraction efficiency, most likely due to the decomposition of formic acid under microwave-assisted conditions. The maximum yttrium yield of 60% was achieved after 5 min of treatment. Nevertheless, microwave-assisted leaching resulted in near-twofold improvement in yttrium recovery compared to conventional leaching. Importantly, the process enabled nearly complete recovery of Nd (100%) and high recovery of Pr (≈92%), while maintaining iron dissolution below 4%, indicating a high level of selectivity toward rare earth elements.

**Figure 8:**
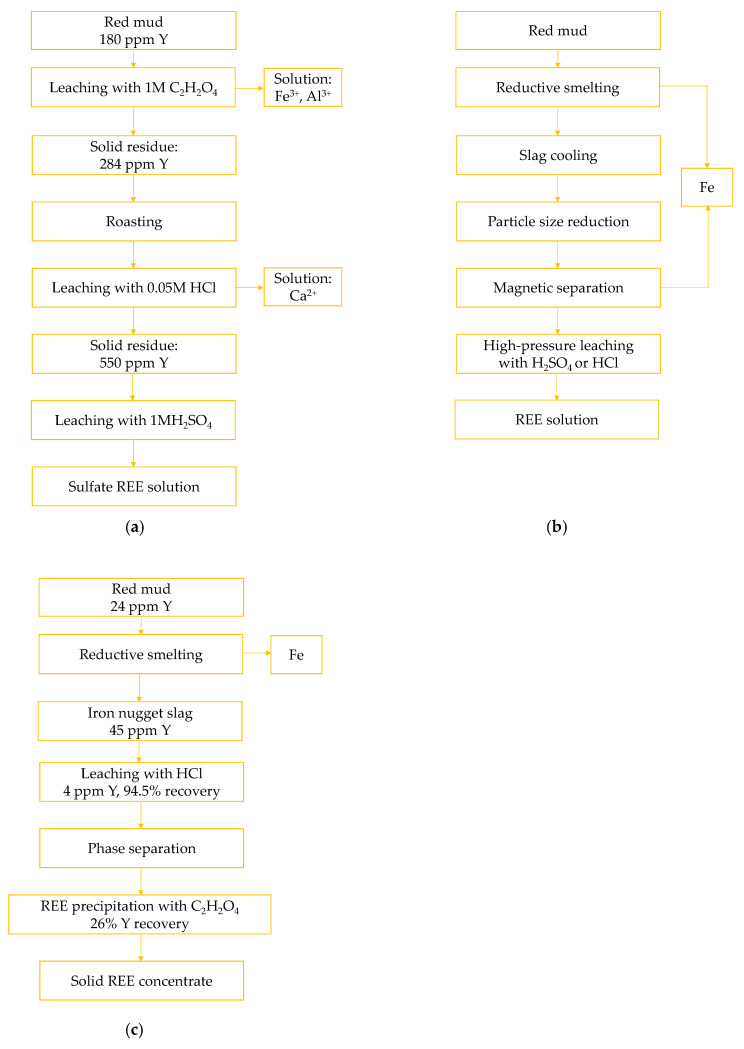
Schemes of yttrium recovery from red mud by combined pyrometallurgical and hydrometallurgical routes. Adapted from: (**a**) [154], (**b**) [157], (**c**) [158].

Pre-leaching procedures have likewise been developed to reduce the dissolution of impurity ions (e.g., Fe, Al, Ca), which subsequently complicate REE separation and purification. Başturkcu [153] proposed a two-step process in which the initial leaching stage was carried out using H_2_SO_4_ solution to remove up to 75–97% of Na^+^, Ca^2+^, K^+^, and Al^3+^ species. This treatment reduced the pH from 10.5 to 2–4. The final pH proved to be particularly important, as it strongly affected yttrium losses, which reached 8–12% at pH 2–3. These losses could be avoided at pH 4, but under this condition the removal efficiency of impurities decreased drastically (to 10–90%, depending on the element). The solid residue enriched in yttrium was subsequently subjected to a second leaching step with H_2_SO_4_, achieving 40–92% yttrium extraction at leaching temperatures of 25–80 °C. Under the optimized conditions, the overall yttrium recovery reached 84%. Li et al. [154] proposed a modified process in which two leaching stages were separated by a roasting step (Figure 8a). During the initial leaching with oxalic acid, iron and aluminum were selectively removed without significant losses of REEs, which remained concentrated in the solid residue. The obtained residue was subsequently roasted (520 °C) and washed with diluted HCl (L/S 100) to remove calcium ions. In the final stage, the purified solid was leached with H_2_SO_4_, resulting in yttrium recovery of approximately 80–85% at acid concentrations of 3.5–4 M. Importantly, the dissolution of impurity elements into the solution remained below 20%.

Combined pyrometallurgical–hydrometallurgical treatment of red mud appears to be a promising route for improving yttrium leachability. Borra et al. [155] mixed red mud with concentrated H_2_SO_4_ to convert metals into their sulfates, followed by drying and a roasting (650–700 °C) to transform iron sulfate into Fe_2_O_3_. Importantly, the roasting time should not exceed 2 h, as longer treatment led to a decline in subsequent extraction efficiency. The roasted product was then subjected to water leaching under both agitated and non-agitated long-term conditions, resulting in high overall REE recovery, including approximately 90% Y and 95% Dy, while Sc recovery reached about 60%. In contrast, iron dissolution remained below 1%, indicating a highly selective separation of rare earth elements from the treated residue. In turn, Rivera et al. [157] compared the water leachability of red mud after dry digestion with either H_2_SO_4_ or HCl. The results demonstrated that HCl acid was the more effective reagent, while yttrium concentrations in chloride solutions were near three times that obtained in sulfate media. The proposed two-step process effectively eliminated silica polymerization, thereby enhancing metal extraction efficiency. Interestingly, conducting the process in multiple sequential stages led to a gradual increase in REE concentrations in the chloride solution, to a much greater extent than in the sulfate system. Importantly, lower iron concentrations were simultaneously observed in the leachates, indicating improved selectivity of the chloride-based process.

Further studies [156,158] incorporated a smelting reduction stage aimed at iron recovery while retaining REEs within the slag phase. The resulting slag was subsequently leached with inorganic acids, either sulfuric or hydrochloric, to produce a final REE-bearing solution [156], or alternatively the dissolved REEs were precipitated with oxalic acid to obtain a solid REE concentrate [158]. Leaching of REEs from the slag under high-pressure conditions proved to be highly effective in hydrochloric acid (3 M), with extraction efficiencies exceeding 95%, whereas sulfuric acid resulted in recovery below 20% [156]. The application of more concentrated HCl solutions (12 M) enabled nearly 95% yttrium extraction at 90 °C under ambient pressure [158]. However, the subsequent precipitation step as oxalates resulted in a much lower overall recovery, reaching only approximately 26%.

### 4.3. Solvometallurgical Treatment

The application of solvometallurgical approaches to red mud processing still appears to be at an early stage of development [159,160]. Davris et al. [159] investigated the direct leaching of red mud (115 ppm Y, 10% of the total REE) using the functionalized ionic liquid betainium bis(trifluoromethylsulfonyl)imide HbetTf_2_N (90 °C, 24 h). Overall REE recovery was relatively low, remaining below 50%, with yttrium extraction reaching 43%, while calcium was dissolved almost completely. Importantly, less than 5% of Fe and only about 2% of Al were transferred into the leaching solution, indicating relatively high selectivity toward REEs. The addition of 4% water significantly improved the extraction of most REEs to 65–85%, including nearly 70% yttrium recovery, whereas the dissolution behavior of the major matrix elements remained largely unchanged.

Rüşen et al. [160] explored the applicability of deep eutectic solvents based on choline chloride systems. Although yttrium was reported to be present in the red mud (60 ppm), the behavior and recovery of this element were unfortunately not discussed in the study.

### 4.4. Biohydrometallurgical Treatment

Biohydrometallurgical methods have been considered a potential alternative for red mud treatment due to the relatively low concentrations of valuable metals present in the residue. In practice, however, these methods generally exhibit rather limited REE leaching efficiencies, typically below 60% [161]. Van Wyk et al. [162] reported that *Gluconobacter oxydans* promoted dissolution of red mud more effectively than several conventional acids (acetic, citric, gluconic, oxalic, nitric, hydrochloric, and sulfuric). Nevertheless, the leaching efficiencies for most investigated REEs remained relatively low, reaching 13% for Sc, 15% for La, 24% for Ce, and 11% for Nd. Against this, yttrium exhibited high leachability, with recovery reaching 41%. Qu and Lian [163] developed two different bioleaching strategies using *Penicillium tricolor*: (1) direct incubation of the fungus with red mud, and (2) a two-step process involving fungal preculturing in sucrose medium followed by the addition of sterilized red mud. Both approaches proved effective, with yttrium again identified as the most readily leached REE, achieving recovery of 65–75%. Interestingly, increasing pulp density (2–10%) produced opposite trends for the two methods: in the direct incubation approach, yttrium recovery decreased with increasing pulp density, whereas in the two-step process the recovery improved, although yttrium consistently remained the best-leached REE. In turn, Cozzolino et al. [164] investigated the bioleaching potential of microbial biomass naturally present in red mud (199 ppm Y). Under these conditions, yttrium extraction reached 30% compared with 20% for Ce and La, 34% for Sc, and as much as 65% for Nd.

## 5. Yttrium Recovery from Coal, Coal Gangue and Coal Ash

### 5.1. General

**Figure 9:**
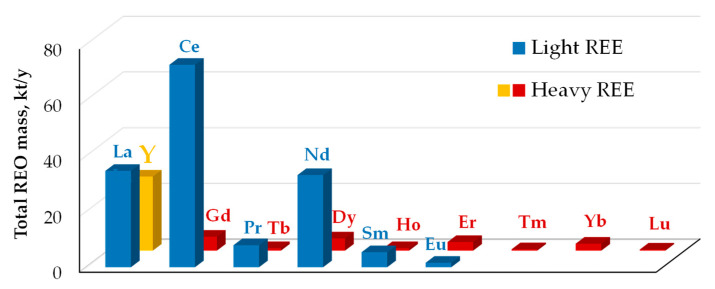
Estimated mass of REE oxides REO in coal ash discarded in China (2019). Adapted from [166].

Coal, coal gangue (solid waste generated during coal mining, accounting for 15–20% of total raw coal production), and coal ash (fine-grained solid residues formed during coal combustion) are carriers of trace amounts of valuable metals (e.g., Au, Ga, Ge, REEs) [165]. Particularly REE-enriched coal deposits have been identified in Russia (Far East, 5952 ppm), China (Guizhou, 2491 ppm; Chongqing, 1264 ppm; Guangxi, 1095 ppm), the United States (Kentucky, 1460 ppm), and Tajikistan (Nazar-Ailok, 1836 ppm) [166]. Ketris and Yudovich [165] reported the average abundance of REEs in world coals to be 68.6 ppm, which may correspond to about 50 million tons of global reserves, a quantity comparable to that of REE-bearing ores [166]. Coal ashes are even more enriched in REEs due to the removal of the carbonaceous fraction during combustion, with the average concentration estimated at 435.5 ppm [165]. However, it should be emphasized that the distribution of individual elements in these materials is not uniform, with cerium appearing to be the dominant rare earth element occurring (23 ppm in coal, 130 ppm in coal ash [165]). Considering the global scale of coal consumption (about 8800 Mt/y in 2024–2025 [167]) and the substantial generation of waste products (600–800 Mt/y of fly ash [168] and 780 Mt/y of bottom ash [169]), these materials are suggested [170,171] as alternative sources of REEs (Figure 9).

Yttrium concentrations in coals and coal by-products depend on their source region (Table 9). Although global average element concentrations are similar in both brown and hard coals (mean 8.4 ppm), some differences are observed in their ashes (mean 51 ppm), resulting from variations in the combustion of carbonaceous components. Interestingly, the share of yttrium in the total REE content in ashes remains relatively comparable, although it tends to increase in the case of ashes produced from REE-enriched coals.

The recovery of metals from coal and its by-products is challenging not only due to their relatively low concentrations but also because of their complex phase composition. Following the combustion of carbonaceous coal components, coal fly ash and bottom ash are predominantly composed of silicate and aluminosilicate glass, along with mineral phases and residual carbonaceous combustion fragments [168,174,175]. Coal gangue is primarily composed of minerals such as quartz, kaolinite, pyrite, boehmite, and mica [179,180]. The chemical composition of all these waste materials is predominantly dominated by oxides: SiO_2_, Al_2_O_3_, Fe_2_O_3_ and CaO.

The occurrence mode of REEs in these materials is variable and influenced by many factors, making it difficult to reach a general consensus [166,170,171]. However, through sequential extraction procedures, it has been generally observed that yttrium tends to accumulate in the aluminosilicate residual (glass) fraction of these materials in proportions ranging from 50% to 90% [166,182], while metal oxide with organic matter bounded forms can be also dominant in coal gangue [183].

### 5.2. Coal

Although direct metal recovery from coals does not seem rational, there are several examples that highlight the potential of acid leaching processes. For instance, Laudal et al. [178] compared the leachability of REEs from lignite coals. They found that low-concentration (0.1 M) inorganic acids were ineffective, with recovery below 10%. However, more concentrated acids produced significantly better results, with yttrium recovery increasing in the following order: 1 M H_3_PO_4_ (~75% Y) < 0.5 M H_2_SO_4_ (~85% Y) < 1 M HCl (~92% Y). Interestingly, yttrium was found to be relatively well soluble compared to other REEs.

Zhang and Honaker [184] investigated the float fractions of bituminous coals from different sources. To completely remove the organic matter, the samples were calcined (600 °C, 2 h) before leaching. It was found that calcination altered the occurrence modes of HREEs, shifting them from predominantly metal oxides, insoluble, and acid-soluble forms to ion-exchange and carbonate fractions. This transformation allowed for more effective leaching under mild conditions using an ammonium sulfate solution (1 M pH 4, 75 °C). As a result, yttrium recovery reached 60–85%, the highest among all REEs, which typically had recovery below 50%.

Unconventional methods were applied by Haque et al. [185] to recover REEs from low-ash subbituminous coal (10 ppm Y). They compared three methods: (1) extraction with ethanol or toluene at their boiling temperatures, (2) conventional acid leaching (90 °C) with acid mixture of iron sulfate salts followed by solvent extraction with ethanol or toluene, and (3) coal electrolysis (90 °C) conducted in a two-compartment electrolyzer (iron(II,III) sulfate as anolyte, H_2_SO_4_-based coal slurry as catholyte) separated by an ion-exchange membrane. Yttrium extraction (as well as other REEs) with organic compounds was only 10%, while acid leaching and electrolysis improved extraction to near 35% and 45%, respectively. However, none of the methods significantly improved subsequent solvent extraction yield (5–15%) compared to direct extraction from coal using organic compounds.

### 5.3. Coal Gangue

The recovery of metals from coal gangue not only facilitates the high-value utilization of waste but also helps prevent the shortage of mineral resources. However, even leaching with HCl results in poor recovery rates (below 30%) for HREEs, while the recovery of LREEs is about twice as high [186]. This is due to the higher concentration of HREEs in the residual fraction composed aluminosilicate–silicate matrix. Therefore, the primary proposed solution for releasing these elements is roasting of coal gangue [179,181,183].

**Figure 10:**
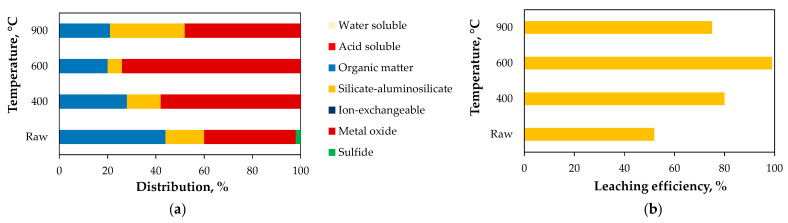
Effect of calcination temperature on yttrium: (**a**) occurrence mode in coal gangue; (**b**) leaching efficiency in 3 M HCl (2 h). Adapted from [183].

Pan et al. [183] showed that calcination at an optimal temperature of 600 °C doubles the accumulation of HREEs in the metal oxide fraction to 70% while reducing their presence in the organic phase to 15%. When considering yttrium behavior individually, similar changes were observed (Figure 10a). These changes resulted from the conversion of kaolinite and boehmite into metakaolin, which is stable at 600 °C, while further temperature increases transform the latter into an amorphous phase. The changes in the phase composition govern yttrium leachability (in HCl), which increased from about 50% for raw coal gangue to nearly 100% at a calcination temperature of 600 °C (Figure 10b). This behavior was also confirmed by Chen et al. [178], who validated the optimal calcination temperature of 600 °C. However, their study primarily discussed the behavior of both REE subgroups without specifically addressing yttrium. On the other hand, Ji et al. [180] investigated various acids, including HCl and organic acids (acetic, L-ascorbic, maleic, DL-malic, malonic, oxalic, succinic, DL-tartaric, citric), for the leaching of calcined waste. Unfortunately, only the overall leaching efficiencies of HREEs were reported, which were lower (up to 35%) compared to LREEs (up to 75%).

### 5.4. Coal Fly Ash

Coal fly ash appears to be the best by-product for recovery, as it is produced in large quantities, representing 40–90% of the total combustion residues [168]. However, despite the considerable number of publications related to REE recovery, only some specifically discuss the leachability of yttrium. Given that this element is bound in weakly soluble phases [177], their leaching under mild conditions results in low yields, while HCl appears often to be the selected leaching agent (Table 10). Pan et al. [187] showed that efficiency of HREEs (and the whole REE group, in fact) leaching in acids was 16–20% and it decreased in order HCl > H_2_SO_4_~HNO_3_ (approximate results for Y: 18% in HCl, 16% in H_2_SO_4_, 14% in HNO_3_). Bartoňová et al. [177] showed that in coal combustion ashes, yttrium is predominantly associated with phosphorus and titanium oxides. Following treatment with diluted HCl (2:1), the relationship with P_2_O_5_ weakened significantly, leaving TiO_2_ as the primary host mineral for yttrium. This hindered its release, resulting in a modest yttrium extraction of only 50%.

As REEs accumulate in the fine-grained and non-magnetic fractions of fly ash [188], physical separation methods have proven effective as a preliminary step for the concentration of these elements. Therefore, a combination of size classification and magnetic separation was shown to significantly improve yttrium extraction with HCl, nearly doubling the recovery to 80%.

Tang et al. [189] introduced alkali fusion (860 °C, Na_2_CO_3_) prior to acid leaching to convert aluminosilicates into acid-soluble compounds. This significantly increased yttrium leaching with HCl to about 45% compared to the direct untreated ash, which yielded only around 5%. Process optimization through careful selection of fusion temperature, solid-to-liquid ratio, acid concentration, and agitation rate led to a significant improvement, achieving a leaching efficiency of 85%. Notably, yttrium was much more easily leached, with about 20% higher recovery compared to other REEs (La, Ce, Pr, Nd).

In turn, Liu et al. [190] compared the leachability of two coal fly ashes from different coal origins, but with relatively comparable yttrium content. They found that bituminous ash was more resistant to the action of citric acid than subbituminous ash, resulting in a significant difference in yttrium extraction, reaching nearly 60%. These differences were attributed to the phase composition, which was also reflected in the content of the main components (class F and C). The leached REEs were then recovered by precipitation with oxalic acid, resulting in the formation of REE concentrates with a 2- (to 90 ppm) and 4- (to 199 ppm) fold increase in yttrium content in ash originated from coals of class F and C, respectively.

**Figure 11:**
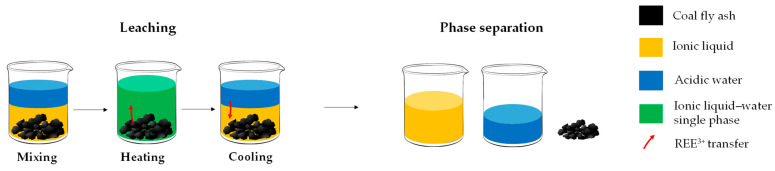
Leaching of coal fly ash with water-saturated ionic liquid. Adapted from [191].

The extractability of REEs using water-saturated (acidified) ionic liquid HbetT_2_N from pretreated fly ash (NaOH, 85 °C) was also investigated [191,192]. After the extraction stage (Figure 11), yttrium was distributed approximately 50% in the aqueous phase, 28% in the ionic liquid phase, while the rest remained in the solid residue [191]. The leaching efficiency of yttrium reached 80 ± 5%, and was only slightly influenced by the leaching time (0.5–12 h), temperature (45–85 °C), and pH of the aqueous phase (2–7). Interestingly, under these conditions, the extraction of impurities was variable, but allowing for the selective separation of REEs from Fe, Ti, Si, Mg, and Ca to some extent. Notably, the recovery of metal ions from loaded ionic liquid phase could be simple achieved by stripping with HCl solution [191,192].

## 6. Yttrium Separation from Solutions

Leaching of yttrium-bearing waste materials typically generates leachates with yttrium concentrations below 1 g/L. Considering that yttrium ions exhibit strong chemical similarities to the other accompanying REE^3+^, they tend to precipitate together as oxalates. This is of course the simplest method, as it results in an REE concentrate relatively free from the major metallic impurities, which are present in the leachates at concentrations of several grams per liter at a minimum. The separation of REEs from oxalates, which are then converted into oxide concentrates, appears to be a relatively straightforward process, as these methods are commercially implemented. Thus, conventional transformation to halides (chlorides, fluorides) followed by molten salt electrolysis or carbothermic reduction are well-established techniques [193], despite of high costs of high-temperature methods. Consequently, yttrium solvent extraction has gained attention as an alternative approach to produce high-purity yttrium oxide, such as the application of naphthenic acid (separation from lanthanides), P507 (separation from lanthanum and calcium), and Aliquat 336 (separation from iron, zinc, and lead) in a multistage process [194].

There are two main research trends in the application of solvent extraction for yttrium separation. These involve the use of traditional organic solvents or ionic liquid extractants, with fewer studies focusing on the use of deep eutectic solvents. Some examples of developed systems are shown in Table 11.

Most of the solvent extraction processes are investigated using synthetic solutions, which is understandable, as optimal conditions need to be determined. However, there are very limited data on the performance of these processes in real-world systems, where a range of impurities, including base metals, may be present at high concentrations. This requires special attention, as the presence of such impurities can significantly affect the selectivity and efficiency of the extraction process. In real systems, competitive extraction of base metals may lead to co-extraction with yttrium, requiring additional purification steps or modification of the solvent system to achieve high purity. Therefore, further research is needed to adapt and optimize these processes for large-scale applications, considering the complexity and variability of real feedstocks.

## 7. Summary Remarks

Yttrium is a lesser-known member of the rare earth element group, but it has recently attracted attention due to its extraordinarily high price rise, which has surpassed even those of terbium and dysprosium, two of the most critical metals. These price fluctuations stem from the highly centralized nature of its extraction and production, which in turn impacts its availability on global markets. However, the recovery of yttrium from other sources worldwide could potentially address the issue of availability, provided that yttrium-bearing waste is managed properly.

A review of the literature indicates that various secondary materials, such as spent phosphors, phosphogypsum, red mud, and coal combustion fly ashes, are currently the most important sources for prospective yttrium recovery. However, with further exploration, other materials like spent zirconia, alloys, acid mine drainage could certainly be identified as possible sources. Despite the widespread availability of different waste materials, recovering yttrium remains a challenge. The primary difficulty lies in the trace concentrations of the element and the complex, multicomponent nature of the wastes (Table 12). This complexity involves not only major metal impurities but also the similarity of yttrium’s properties to those of the entire REE group.

Spent phosphors show the most promise for solvent-based recycling due to their relatively simple composition compared to the other materials discussed. However, despite the relatively high yttrium content in phosphors, their low fraction in the overall waste stream requires the processing of large quantities of waste materials to obtain sufficient amounts of yttrium-bearing phosphor material. This additionally involves the separation of phosphors from other waste components, such as glass, plastics and other fractions. From a high-yttrium-content perspective, red mud is more attractive, despite its problematic high alkalinity and high iron concentration. In contrast, coal fly ashes, although widely available, bind yttrium in hardly soluble phases, necessitating pretreatment to enhance the release of the desired element. Phosphogypsum is also increasingly recognized as a viable source, as evidenced by large-scale tests for the recovery of the most important REEs.

**Figure 12:**
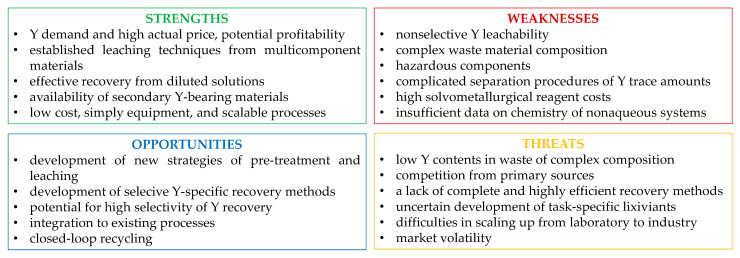
SWOT analysis of yttrium recovery from secondary materials.

Traditional hydrometallurgical methods have been widely confirmed and are the most likely to be implemented in practice. They most commonly employ strong inorganic acids as leaching agents, which, although effective, are non-selective and require specific methods for separating yttrium (and other REEs) from the major elemental components. These latter constituents, however, should not be neglected in the development of recovery processes. More innovative organic systems (solvometallurgical), although considered green alternatives, have been tested in a very limited scope, and their large-scale implementation is rather unlikely. Bioleaching, though an environmentally friendly route, does not yet appear to be sufficiently effective at this stage to warrant large-scale implementation. Although initial research appears promising, the shift from laboratory-scale experiments to large-scale, long-term industrial applications remains a significant barrier (Figure 12).

It should be emphasized that results obtained at the laboratory level do not necessarily translate into economic feasibility at industrial implementation. Over the years, several economic assessments of yttrium recovery from some types of the waste discussed herein have been reported [40,46,204], indicating that process profitability is generally achievable only under conditions of sufficiently high yttrium prices (e.g., above 14 EUR/kg Y for FLs recycling in 2019 [40]), adequate recycling scale, and availability of suitable feedstock. The latter is essential to ensure process continuity and requires the establishment of a well-organized supply chain for secondary materials. At the same time, the highly dynamic fluctuations in metal and energy prices do not provide a stable basis for long-term economic predictability, which complicates the development of consistently profitable recycling strategies. Although financial risk mitigation mechanisms exist to address such variability, their application requires careful and case-specific evaluation. Nevertheless, promising opportunities may arise from the further development of recovery methods focused not only on maximizing the extraction of yttrium with valuable co-occurring elements, but also on integrating yttrium recovery processes with existing rare earth element separation systems. Such approaches contribute to the diversification of critical element supply sources and help reduce dependence on supply chains vulnerable to geopolitical conditions.

These factors undoubtedly create opportunities to drive scientific progress and foster innovative solutions to complex recovery challenges. Enhancing pretreatment methods and optimizing extraction techniques could significantly improve yttrium recovery from secondary sources. Additionally, the development of new extracting systems for selective separation could effectively separate yttrium from other ionic impurities, increasing process efficiency. By refining these approaches, there is potential to establish more sustainable and cost-effective processes, which could ultimately facilitate greater scalability and broader industrial adoption. However, achieving this will require development of advanced closed-loop systems and substantial financial investment and effort.

## Figures and Tables

**Table 1:** Yttrium concentration in primary rare earth sources worldwide.

Deposits	Main Host Mineral	Y_2_O_3_ Concentration, wt%	Y_2_O_3_ in Total REOs *, %	Ref.
Australia, Browns Range	xenotime-Y	0.357	56.68	[21,24]
Australia, Mount Weld Duncan	carbonatite laterite	0.250	5.17	[21,24]
Australia, Nolans Bore	fluorapatite	0.035	1.35	[21,24]
Canada, Strange Lake Enriched	kainosite, Y–Ca silicate	0.470	32.62	[21,25]
China	ion-adsorption clays	–	24.6	[26]
Greenland, Kvanefjeld	steenstrupine, eudialyte	0.084	7.70	[21,27]
Kenya, Mrima Hill	carbonatite laterite	0.209	2.97	[21,28]
Namibia, Lofdal	xenotime–Y, carbonatite	0.341	57.70	[21,28]
South Africa, Steenkampskraal	monazite, apatite	0.579	4.13	[21,28]
Sweden, Norra Kärr	eudialyte, catapleiite	0.218	35.98	[21,29]
United States, Mountain Pass	carbonatite, bastnasite	0.007	0.10	[21,30]
United States, Round Top	fluorite	0.028	43.90	[21,31]
Global REE deposit average *	–	–	3.30	[32]
Continental crust (total)	–	0.0019 Y	–	[33]

* Based on 59 global REE deposits [31].

**Table 2:** Yttrium concentration in phosphor-containing wastes.

Waste Type	Concentration, wt%		Ref.
	Y	Y_2_O_3_	
Compact fluorescent lamps	0.254	–	[50]
Tubular fluorescent lamps	0.423	26.4	[55,56]
Fluorescent lamps (mixed)	0.680	–	[57]
Fluorescent lamps (after Hg evaporation)	8.4–9.3	–	[58,59,60]
Fluorescent powders (tricolor)	10.3	40	[61,62]
LED bulbs	0.00017	–	[50]
LED modules	0.0001–3.41	–	[49,56,63,64,65,66]
Cathode ray tube powders	3.43–18.10	–	[67,68,69,70,71,72]

**Table 3:** Yttrium recovery methods from spent FL phosphors.

Material	Pretreatment	Leaching Conditions	Leaching Efficiency	Further Stages; Recovery	Ref.
milled TFLs (180 μm), 0.42% Y	–	2 M H_2_SO_4_, 65 °C, 5 h, S/L 0.25 g/L	44%	Sorption on D2EHPA-impregnated resin; 90%	[55]
spent FL powder (d_90_ = 29 μm), 15.8% Y	–	2 M H_2_SO_4_, 80 °C, 1 h, S/L 5%	~100%	Oxalate precipitation; ~100%	[80]
grinded FLs, 6.8% Y	–	0.5–4 M HNO_3_, 20 °C, 168 h, S/L 10%	97%	SX: Cyanex 923/4 M HCl	[78]
		0.5–4 M HCl, 20 °C, 168 h, S/L 10%	98.2%	–	
		0.5–4 M H_2_SO_4_, 20 °C, 168 h, S/L 10%	98%		
		1 or 4 M CH_3_SO_3_H, 20 °C, 168 h, S/L 10%	98%		
milled TFLs (<75 μm), 19.5% Y	halo phosphate leaching in HCl	2 M HCl, 80 °C, 1 h, S/L 100 g/L	~100%	Double sodium yttrium sulfate precipitation (>99% purity)	[81]
waste FL phosphor (<45 μm), 14.6% Y	–	4 M HNO_3_, 80 °C, 1 h, S/L 10%	14.8 g/L	CaSO_4_ precipitation, SX: D2EHPA/6 M HCl, oxalate precipitation	[82]
crushed TFL phosphor, 24.4% Y_2_O_3_	alkali mechanical activation	HNO_3_	0.04 M	Oxide electrodeposition (91% purity)	[56]
milled FLs (<200 μm), 3.2% Y	–	2 M C_6_H_8_O_7_, 90 °C, 2 h, S/L 5%	87%	Oxalate precipitation; 99%	[83]
		4 M CH_3_COOH, pH 0, 90 °C, 0.5 h, S/L 5%	100%	Oxalate precipitation; 6%	
		2 M C_2_H_5_NO_2_, pH 2, 90 °C, 2 h, S/L 5%	79%	Oxalate precipitation; 100%	
		2 M HNO_3_, 90 °C, 2 h, S/L 5%	95%	Oxalate precipitation; 36%	
milled FLs (10 μm), 10% Y	–	3.25 M HCl, 160 °C MW, 1.5 h, S/L 3%	95%	–	[62]
		3.25 M H_2_SO_4_, 160 °C MW, 1.5 h, S/L 3%	96%		
		3.25 M HNO_3_, 160 °C MW, 1.5 h, S/L 3%	95%		
milled FLs (100 μm), 9.3% Y	–	2 M H_2_SO_4_, 5% H_2_O_2_, 60 °C, 1 h, S/L 5%	90%	–	[60]
	alkali fusion		99%		
milled FLs, 8.6% Y	alkali fusion	2 M HNO_3_, 60 °C, 0.4 h, S/L 30 g/L	89%	–	[59]
milled FLs (d_50_ 2 μm), 33% Y	solid-state chlorination	1 mM HCl, 25 °C, 0.5 h	90%	SX: Cyanex 923/4 M HCl, oxalate precipitation (94% purity)	[84]
milled FLs (<125 μm), 13.3% Y	halo phosphate leaching in CH_3_SO_3_H	5% CH_3_SO_3_H, 80 °C, 2 h	100%	SX: D2EHPA/6 M HCl, oxalate precipitation	[85]
milled FLs	–	2 M H_2_SO_4_, 5% H_2_O_2_, 80 °C, 2 h, S/L 15%	0.5 g/L	ATPS: L64 + alizarin red + H_2_O + Na_2_SO_4_; 90%	[86]

**Table 4:** Efficiency of yttrium leaching in choline chloride–malonic acid deep eutectic solvent at optimal operation parameters [91,92].

Factor	Leaching Type			
	Mechanical Oscillation	Bottom-Focused Microwave	Ultrasound	Focused Ultrasound
Parameter	800 rpm	20 W	240 W	240 W
Mechanical Activation	no or yes	yes	no	no
Water Content in DES	0 or 7.5%	7.5%	0%	0%
Efficiency, %	30.6 (90 °C) or 52 (100 °C)	95.7	35.7	90.1
Time, h	12	1.5	12	1

**Table 5:** Yttrium concentration in phosphogypsum.

Source Region	Y Concentration, ppm *	Y Share of Total REE, %	Ref.
Brazil (Santa Catarina)	98.8 ± 2.7	1.9	[108]
Canada (Alberta)	53	2.6	[109]
China (Guizhou, Yunnan)	20.9/74	25/36	[104,107]
Finland (Yara Siilijärvi)	31.8	–	[110]
Morocco (Ouled Abdoun, Gantour, Jorf Lasfar)	55–163	35–41	[111,112]
Philippines (fertilizer plant ponds)	69.7 ± 35.2	26	[113]
Poland (Wizów)	192 ± 22	–	[114]
South Africa (Phalaborwa)	77.7	2.5	[115]
Spain (Huelva)	119	37	[116]
Tunisia (Sfax, TCG Mdhilla Gafsa)	54–85	20–23	[117,118,119]
USA (Florida)	34	–	[120]

* 1 ppm = 0.0001%.

**Table 7:** Yttrium concentration in red mud.

Red Mud (Type) Source Region	Bauxite Deposit Type	Y Concentration in Red Mud, ppm	Y Share of Total REE, %	Ref.
China (high-iron diaspore)	–	252	18	[143]
China (low-iron diaspore)	–	84	9	[143]
France	Lateritic	118–123	16	[144]
France	Karstic	184–265	1–12	[144]
Greece	Karstic	108 ± 2	11	[145]
Greece	Lateritic + Karstic	76 ± 10	8	[146]
Guinea	Lateritic *	101 ± 6	18	[144]
India	–	170 ± 10	19	[147]
Italy	–	88	16	[148]
Iran	–	44	–	[149]
Jamaica	–	373 ± 4	27	[147]
South Korea	–	39 ± 4	12	[147]
Turkey	Karstic	200	–	[150]
Turkey	–	32–145	5–10	[151]
USA	–	46 ± 12	14	[147]

* 34 ppm Y, 16% of total REEs.

**Table 8:** Yttrium leaching from red mud.

Y Content, ppm	Pretreatment	Leaching Conditions	Leaching Efficiency, %	Ref.
76	–	6 M HCl, 25 °C, 24 h, S/L 5%	80	[146]
60	–	3 M HNO_3_, 95 °C, 8 h, S/L 3%	98	[152]
		4 M HCl, 75 °C, 8 h, S/L 3%	100	
126	Leaching with H_2_SO_4_ (to pH 3)	H_2_SO_4_, 90 °C, 1.5 h, S/L 40%	84	[153]
180	Leaching with C_2_H_2_O_4_; roasting; leaching with HCl	1 M H_2_SO_4_, 95 °C, 3 h, S/L 20%	70	[154]
44	–	0.6 M HCOOH, MW 600 W, 5 min, S/L 10	60	[149]
76	Sulfation with H_2_SO_4_; roasting	H_2_O, 25 °C, 7 days, S/L 2%	90	[155]
66	Reductive smelting in EAF *	3 M HCl, 120 °C, 1 h, S/L 10%	98	[156]

* EAF—electric arc furnace.

**Table 9:** Yttrium concentration in coal and its by-products.

Source Region	Type *	Y Concentration, ppm	Y Share of Total REE, %	Ref.
Coal				
World	Lignite	8.6 ± 0.4	12	[165]
World	Bituminous	8.2 ± 0.5	12	[165]
India, Gondwana Coalfield	(Sub)bituminous	14–46	11–20	[172]
India	Lignite to sub-bituminous	0.3–24.2	1–13	[173]
Indonesia, Java	Sub-bituminous	0.5–4.4	16–21	[174]
Turkey, Kangal	Lignite	5.3–11	12	[175]
USA, North Dakota	Lignite	84–153.6	14	[176]
Coal Ash				
World	Lignite	44 ± 3	10	[165]
World	Bituminous	57 ± 2	13	[165]
China, Guangxi	–	121–296	11–25	[171]
China, Songazo	–	97–462	11–23	[171]
China, Yunnan	–	95–189	7–15	[171]
Czech Republic	Lignite: fly ash/bottom ash	31/34–53	–	[177]
India, Gondwana Coalfield	(Sub-)bituminous	21–124	11–13	[172]
Indonesia, Java (power plant)	Sub-bituminous: fly/bottom	34–46/26–47	17–19	[174]
Poland, power plants	Bituminous: fly ash	40–73	13–15	[175]
Poland, power plants	Lignite: fly ash	18–63	10–18	[175]
Russia, Pavlovka	–	197–3540	22–42	[171]
Russia, Rakovka	–	179–332	15–20	[171]
Tajikistan, Nazar-Ailok	–	200–800	18–41	[171]
Turkey, Kangal (power plant)	Lignite: fly/bottom	15–21/12–22	8–33	[178]
Coal Gangue				
China, Inner Mongolia	–	42.8	9	[179]
China, Shanxi Province	–	31	9	[180]
USA, Western Kentucky	–	27.6	7	[181]

* Lignite—brown coal, bituminous—hard coal.

**Table 10:** Yttrium leaching from coal fly ash.

Y Content, ppm	Pretreatment	Leaching Conditions	Leaching Efficiency, %	Ref.
110	–	HCl, 60 °C, 2 h	~45	[188]
	Size classification (38 μm)		~60	
	Size classification (38 μm), magnetic separation (nonmagnetic phase)		~80	
57	Alkali fusion	2 M HCl, 2 h	~85	[189]
40	–	HCl	55	[177]
52.8 ± 2.6 *	–	0.1 MC_6_H_8_O_7_ (pH 4), 25 °C, 4 h	12	[190]
44.1 ± 1.6 **	–		70	
105	Alkali treatment	HbetTf_2_N + H_2_O (pH 3.5), 85 °C, 3 h	80 ± 5%	[191]

* Bituminous coal (class F: SiO_2_ + Al_2_O_3_ + Fe_2_O_3_ ≥ 70%). ** Subbituminous coal (class C 50% ≤ SiO_2_ + Al_2_O_3_ + Fe_2_O_3_ ≤ 70%).

**Table 11:** Yttrium separation with solvent extraction.

Solution Type	Extraction/Stripping	Remarks	Ref.
Conventional Extractants			
Chloride	2-hexyldecanoic acid in kerosene/HCl	Extraction sequence: REE > Ce > Y > La; 99.9% purity Y concentrate in cascade SX	[195]
Chloride	Naphtenic acid, trioctyldecylamine, sec-octylalkohol, isopropanol in hexane/–	Two step SX to separate from REE; Y accumulated in organic phase	[196]
Chloride	PC88A in kerosene/HCl, HNO_3_ or H_2_SO_4_	Separation efficiency: HNO_3_~HCl > H_2_SO_4_; HNO_3_ for separation Y from LREEs; H_2_SO_4_ for separation Y from HREEs	[197]
Sulfate			
Nitrate			
Sulfate	Primene JM-T/–	Separation of REEs from Cu; 88% Y extraction (nonselective)	[198]
Phosphate	TOPS 99 in kerosene/HCl, HNO_3_ or H_2_SO_4_	Nonselective SX; Y stripping efficiency: H_2_SO_4_ > HCl > HNO_3_	[199]
Chloride Nd:YAG leachate + PEG 200	Cyanex 272 in 260# solvent oil/HCl	Nd, Y nonselective SX; Nd, Y selective stripping	[200]
Ionic Liquid Extractants			
Chloride, or nitrate	Cyphos IL 104/HNO_3_	Higher efficiency and selectivity for two-component IL system; non-effective stripping with HCl	[201]
	Cyphos IL 104, Aliquat 336/HNO_3_		
Chloride	[N_16_MOP][HAD]/HCl *	Low Y extraction; separation from REEs by Y leaving in aqueous phase	[202]
Deep Eutectic Solvent Extractants			
Chloride	1-decanol, oleic acid, bis(2-ethylhexyl)amine/HCl *	Y selective separation from HREEs	[203]

* Over a dozen systems were composed and tested.

**Table 12:** Assessment of yttrium recovery from waste materials.

Aspect	Phosphors	Phosphogypsum	Red Mud	Coal Ash
Mean Y Concentration	tens of percent *	several dozen ppm	tens to hundreds ppm	several dozen ppm
Main Leaching Agents	inorganic acids	inorganic acids	inorganic acids	inorganic acids
Typical Leachability	90–99%	60–85%	60–98%	50–80%
Leaching Selectivity	no	no	no	no
Main Impurities	Zn, Al	Ca, Fe, Al, Sr	Fe, Al, Ca, Ti	Al, Si, Fe
Other REEs (main)	yes (Eu, Ce)	yes (La, Ce)	yes (Sc)	yes (LREE)
Recovery Remarks	Uneconomical for yttrium alone recovery due to its low concentration and high levels of base elements and other impurities; process profitability depends on recovery of other elements/products and actual metal prices; low cost of leaching agents; high leachate consumption due to non-selective reaction; specific separation methods required (e.g., SX); multiple treatment stages needed			

* In phosphor powder separated from overall waste fraction.

## Data Availability

No new data were created or analyzed in this study. Data sharing is not applicable to this article.
